# Prion-induced photoreceptor degeneration begins with misfolded prion protein accumulation in cones at two distinct sites: cilia and ribbon synapses

**DOI:** 10.1186/s40478-021-01120-x

**Published:** 2021-01-29

**Authors:** James F. Striebel, Brent Race, Jacqueline M. Leung, Cindi Schwartz, Bruce Chesebro

**Affiliations:** 1 Laboratory of Persistent Viral Diseases, Rocky Mountain Laboratories, National Institute of Allergy and Infectious Diseases, National Institutes of Health, 903 South Fourth Street, Hamilton, MT 59840 USA; 2 Research Technologies Branch, Rocky Mountain Laboratories, National Institute of Allergy and Infectious Diseases, National Institutes of Health, Hamilton, MT 59840 USA

**Keywords:** Prion, Prion-like, Ribbon synapses, Retinitis pigmentosa, Alzheimer, Parkinson, Ciliopathy, Scrapie, Necrosis, Apoptosis

## Abstract

Accumulation of misfolded host proteins is central to neuropathogenesis of numerous human brain diseases including prion and prion-like diseases. Neurons of retina are also affected by these diseases. Previously, our group and others found that prion-induced retinal damage to photoreceptor cells in mice and humans resembled pathology of human retinitis pigmentosa caused by mutations in retinal proteins. Here, using confocal, epifluorescent and electron microscopy we followed deposition of disease-associated prion protein (PrPSc) and its association with damage to critical retinal structures following intracerebral prion inoculation. The earliest time and place of retinal PrPSc deposition was 67 days post-inoculation (dpi) on the inner segment (IS) of cone photoreceptors. At 104 and 118 dpi, PrPSc was associated with the base of cilia and swollen cone inner segments, suggesting ciliopathy as a pathogenic mechanism. By 118 dpi, PrPSc was deposited in both rods and cones which showed rootlet damage in the IS, and photoreceptor cell death was indicated by thinning of the outer nuclear layer. In the outer plexiform layer (OPL) in uninfected mice, normal host PrP (PrPC) was mainly associated with cone bipolar cell processes, but in infected mice, at 118 dpi, PrPSc was detected on cone and rod bipolar cell dendrites extending into ribbon synapses. Loss of ribbon synapses in cone pedicles and rod spherules in the OPL was observed to precede destruction of most rods and cones over the next 2–3 weeks. However, bipolar cells and horizontal cells were less damaged, indicating high selectivity among neurons for injury by prions. PrPSc deposition in cone and rod inner segments and on the bipolar cell processes participating in ribbon synapses appear to be critical early events leading to damage and death of photoreceptors after prion infection. These mechanisms may also occur in human retinitis pigmentosa and prion-like diseases, such as AD.

## Introduction

Prion diseases are progressive neurodegenerative diseases which affect humans as well as numerous wild and domestic animal species. These diseases are characterized by vacuolar degeneration of the grey matter and gliosis primarily in grey matter of the CNS. In addition, abnormal aggregation of a host prion protein (PrPC) leads to deposition of a protease-resistant PrP isoform (PrPSc) in the nervous system and other organs, which results in damage by unclear mechanisms [25]. Protein aggregation in prion disease proceeds by a process referred to as seeded polymerization or PrP conversion, where initial small aggregates of PrPSc catalyze extension of protein aggregation to generate additional PrPSc [9].

A similar seeded polymerization mechanism has been noted in several other more common neurodegenerative diseases including Alzheimer’s disease, Parkinson’s disease and tauopathies, but in these diseases, the aggregated host proteins are amyloid beta (Aβ), α-synuclein and tau respectively [4, 11, 26]. Because of the similarities in the protein aggregation process in these diseases and prion diseases, they have been referred to as “prion-like” diseases. Some people have suspected that prion diseases and prion-like diseases might be susceptible to similar therapeutic interventions [22, 51]. Thus, at this time, there is high interest in the mechanisms of pathogenesis of all these diseases.

Prion and “prion-like” diseases are known to cause retinal damage in humans and other species, but each disease affects the retina in unique ways [44]. In Alzheimer’s disease patients, Aβ deposition has been associated with degeneration of the retinal ganglion cell layer, photoreceptors and the retinal pigmented epithelium [3, 45]. Ratnayaka et al. have also shown that Aβ plaques may be a key factor in Age-related Macular Degeneration (AMD). In AD, phosphorylated Tau deposits have also been observed from the outer plexiform layer to the ganglion cell layer [31, 32]. Likewise, abnormal α-synuclein aggregates have been detected in retina of Parkinson’s disease patients, and these are implicated in the degeneration of the nerve fiber layer (ganglion cell axons), ganglion cell body layer and inner plexiform layer [57].

In prion disease, results from our lab and others have confirmed deposition of disease-associated PrP (PrPSc) in human, bovine, primate, ovine, cervid, and rodent retina by RT-QuIC, western blot and/or immunohistochemistry [5, 20, 21, 23, 24, 29, 41, 52, 54, 55, 58, 60]. These studies represent examples from both natural disease (human, elk, deer, sheep) and experimental disease (primate, bovine, sheep, rodent). In these studies, damage appeared to affect primarily photoreceptor rods and cones, and damage to other retinal neuronal populations was not clear [50]. In our previous work, mouse photoreceptors were shown to die mainly by the process of apoptosis which coincided with PrPSc deposition [29, 54].

Previously, defective iron transport has been proposed as a mechanism of damage in prion-infected eye [47]. Since photoreceptors rely heavily on iron-containing enzymes for the biochemical reactions of the phototransduction pathways, disruption of iron transport by prion infection may also play a role in retinal damage. Such a mechanism might explain the selective damage to photoreceptor cells by prions.

Photoreceptors are also damaged in human retinitis pigmentosa, which is a major cause of human blindness resulting in a retinal pathology similar to prion diseases. In some forms of retinitis pigmentosa, microglia are known to become activated by the misfolding of mutant host proteins such as rhodopsin, and microglia have also been suspected to be important in the pathogenic process [40, 46]. In prion diseases, microglia activated by the deposition of aggregated prion protein have also been suspected to be a possible mechanism of pathogenesis [18]. However, in our recent studies using PLX5622 to eliminate microglia in vivo, removal of microglia led to a decrease in survival time due to accelerated brain prion pathogenesis in mice [7]. Thus, microglia appeared to be mainly helpful, not harmful, to the host during prion disease. Furthermore, in our studies of retinal prion disease in mice, elimination of microglia gave similar results, showing accelerated retinal degeneration when microglia were reduced or absent [54]. Müller glial cells are also activated during prion disease but are likely a response to damage rather than the cause of pathogenesis [28, 29, 58]. Thus, in vivo damage induced by prion infection might be due to direct effects of aggregated prion protein on photoreceptor cells rather than indirect effects of activated microglia or astroglia.

In the present paper, we studied direct early events of prion protein deposition in mouse retina following intracerebral prion injection. For these studies, we used confocal and epifluorescence microscopy with detection of multiple targets on the same section to localize PrP in its normal and disease-associated forms, while also observing multiple subcellular components of retinal rods, cones, bipolar cells and horizontal cells. Ultrastructural studies were also done to confirm some of the observations. The results showed early deposition of PrPSc in two distinct, subcellular areas of retina as well as interesting novel events of photoreceptor damage in both these areas which preceded the death of both rods and cones while sparing other nearby cell types.

## Materials and methods

### Ethics statement

All mice were housed at the Rocky Mountain Laboratories (RML) in an AAALAC-accredited facility in compliance with guidelines provided by the Guide for the Care and Use of Laboratory Animals (Institute for Laboratory Animal Research Council). Experimentation followed RML Animal Care and Use Committee approved protocol 2016-042.

### Mice

Retinas used in these experiments were obtained from two strains of mice, C57BL/10SnJ and C57BL/6 J-TgGFP/RFP. 79A scrapie-induced retinal degeneration is very similar in these strains and was characterized in our previous publication [54]. An in-house breeding colony supplied C57BL/10SnJ mice,and C57BL/6 J–TgGFP/RFP were also bred in-house as previously described [54]. The GFP and RFP fluorescence was not important to the questions addressed here. All mice were group housed in transparent cages in a 12 h light (250-300lux) /12 h dark cycle and food and water were available ad libitium.

### Scrapie inoculation model

Scrapie inoculations were carried out as previously described [54]. Briefly, mice (4–6 weeks old) were injected intracerebrally (i.c.) in the left hemisphere with 30 μl of a 1% (wt/vol) dilution of brain homogenate pools from C57BL mice terminally ill from 79A scrapie. Brain homogenates contained 1.0 × 10^5^ ID50 / 30 ul after they were diluted for inoculation in phosphate-buffered balanced saline (PBBS) pH 7.2, supplemented with 2% fetal bovine serum (Hyclone, Logan, UT).

The course of disease, details on the clinical symptoms and retinal degeneration were previously well documented in the mouse strains used in this study in our previous publication [54]. Briefly, in the 79A mouse-adapted scrapie model, mice begin showing clinical signs consistent with scrapie between 105 and 120dpi and reach clinical endpoint disease at approximately 160dpi. Thinning of the retina begins around 118dpi and likely causes blindness by the disease endpoint, though this diagnosis is difficult to determine without conducting further tests. The 79A scrapie strain was previously compared to seven other strains of mouse-adapted scrapie and shown to be the most retinal-tropic [15]. At pre-clinical and clinical time-points, mice were euthanized by isoflurane anesthesia overdose followed by perfusion with 10 ml of saline.

### Immunohistochemistry and Immunofluorescence

For immunohistochemistry and immunofluorescence, eyes were removed, placed in 10% neutral buffered formalin for 3 to 5 days and then processed by dehydration and embedded in paraffin as a single block. Next, 5 μm sections were cut using a standard Leica microtome, placed on positively charged glass slides, and air-dried overnight at room temperature. The following day slides were heated in an oven at 60 °C for 20 min. A Ventana automated Discovery XT stainer was used for deparaffinization, antigen retrieval and immunohistochemical staining.

For immunohistochemical staining of PrP antigens were exposed by incubation in CC1 buffer (Ventana) containing Tris–Borate-EDTA, pH 8.0 for 100 min at 95 °C. Staining for PrP was done using human anti-PrP monoclonal antibody D13 [33] which was obtained from tissue culture supernatants made in our laboratory from CHO cells expressing the D13 antibody construct, which were kindly provided by Dr. R. Anthony Williamson, The Scripps Research Institute, La Jolla, CA. D13 culture fluid was used at a dilution of 1:100 (diluted in PBS with 1% normal goat serum and 0.1% Triton X-100) for 2 h at 37 °C. The secondary antibody was biotinylated goat anti-human IgG at 1:500 dilution (Jackson ImmunoResearch, West Grove, PA.), and avidin-horseradish peroxidase was used with DAB as chromogen (DAB Map kit; Ventana Medical Systems, Tucson, AZ.).

For immunofluorescent staining, antigen retrieval for all targets was performed using a Biocare Medical DC2002 Decloaking chamber with sodium citrate buffer at pH 6.0(0.01 M) for 20 min at 120° C / 20 PSI and cooled to 50 °C. For each of the following steps, 250–300 µl of solution was applied to each slide and covered with a temporary plastic coverslip and incubated for a set amount of time. Tissues were blocked first with a normal donkey serum blocking solution (2% donkey serum, 1% BSA, 0.1% Triton X-100, 0.05% Tween 20 in 0.01 M PBS) for 1 h at room temperature and then in 0.1 M Glycine in 0.01 M PBS for 30 min at room temperature. Primary antibodies (Table 1.) were diluted in donkey serum blocking solution and applied for 1 h at room temperature. Alexafluor (ThermoFisher) secondary antibodies were diluted to 1:250 in donkey serum solution and applied for 1 h. In dual or triple stainings, primary antibodies were applied simultaneously, as were secondary antibodies. After each antibody incubation, slides were washed 3 times in 1X PBS for 10 min. Coverslips were mounted with ProLong Gold with DAPI (Life Technologies) and examined and photographed using an Olympus BX51 microscope/Olympus CellSens software or using a confocal microscope as described below.

**Table 1:** Primary Antibodies used in immunofluorescent staining

Antibody	Specificity (antigen/cell type or structure)	Dilution	Host species	Source
D13	Prion protein	1:100	Human	Ref, Matsunaga
Cone Arrestin	Arrestin 3/Cone photoreceptors	1:100	Rabbit	Millipore, AB15282
Cone Opsin	Red, Green opsins/Cone photoreceptors	1:100	Rabbit	Chemicon, AB5404
Rhodopsin	Rhodopsin/Rod photoreceptors	1:100	Rabbit	Millipore, MABN15
GNAT1	G protein subunit alpha transducin 1 /Rod photoreceptors	1:100	Rabbit	Abcam, ab74059
GNAT2	G protein subunit alpha transducin 2 /Cone photoreceptors	1:100	Rabbit	Thermofisher, PA5-22,340
GLUT1	Glucose transporter 1/Cell membranes of many cell types	1:100	Rabbit	Abcam, ab115730
CtBP2	C-terminal binding protein 2/ribeye protein of ribbon synapses	1:100	Rabbit, mouse	Invitrogen, PA-79086 Santa Cruz, sc-17759
PKCα	Protein kinase C/Rod bipolar cells	1:100	Rabbit, mouse	Invitrogen PA5-17,551, MA1-157
Calbindin	Calbindin/Horizontal cells	1:100	Rabbit	Abcam, ab108404
Rootletin	Rootletin/photoreceptor rootlets	1:50	Mouse	Millipore, ABN1714
Centrin3	Centrin3/cilia and basal bodies in photoreceptor inner segments	1:100	Rabbit	Thermofisher, PA5-35,865
Secretagogin (SCGN)	Cone bipolar cells^a^	1:100	Rabbit	Thermofisher, PA5-30,393

^a^SCGN marks 8 of the 12 subtypes of mouse cone bipolar cells [13, 42]

### Numbers of mice studied

The numbers of animals analyzed at each timepoint are presented in Table 2. Uninfected mice used as controls were of similar age to experimental animals, evidence of age-related retinal changes was not observed in the age range of control animals used.

**Table 2:** Number of retinas analyzed by immunofluorescence at timepoints during disease

Timepoint^a^	Antigen tested^b^											
	PrP (D13)	Cone Arrestin	Cone Opsin	SCGN	CtBP2	PKCα	Calbindin	Rhodopsin^d^	GNAT1	GNAT2	Rootletin	Centrin3
Early (67,82,104)	9^c^	3	3	3	3	2	1	3	1	1	3	3
Mid (118, 125, 129, 131)	8	3	2	2	2	3	2	3	1	nd	1	2
Late (144, 153, 159, 163, 165)	8	2	nd	2	3	2	1	2	3	nd	nd	1
Uninfected	5	2	3	2	2	1	1	2	2	1	2	1

*nd* not done

^a^Timepoints are shown in days post inoculation (dpi) with 79A mouse adapted scrapie. In the 79A mouse-adapted scrapie model, mice begin showing clinical signs consistent with scrapie around 105-120dpi and reach clinical endpoint disease at approximately 160dpi. Thinning of the retina begins around 118dpi and likely causes blindness by the disease endpoint.

^b^Antigens detected with antibodies described in Table 1

^c^Number of mice tested with each antibody at timepoint range shown

^d^Data not shown

### Nomenclature and detection of PrP, PrPC and PrPSc

Monoclonal antibody D13 was used in immunostaining of tissue sections to detect PrP. In tissues of uninfected mice, PrP detected was assumed to be the normal PrP isoform, PrPC. In infected tissues, PrP detected in locations different from those seen uninfected mice was assumed to be disease-associated PrPSc, and PrP detected in similar locations to those found in uninfected mice was assumed to be either or both isoforms.

### Quantification of bipolar and horizontal cells

To quantify rod bipolar cells throughout the timecourse of disease, two sections of retina from a mouse at each timepoint were stained with DAPI, anti-PKCα primary antibody and secondary antibody Alexa Fluor 488 as described above. The PKCα-positive rod bipolar cell bodies were counted in four 20X fields per timepoint and averaged. Horizontal cell numbers were determined by staining retinal sections with DAPI, anti-calbindin primary antibody and Alexa Fluor 488 secondary antibody as described above. Calbindin-positive cell bodies were counted along two entire retinal sections from one mouse per timepoint. Cone bipolar cells were counted by staining retinal sections with anti-secretagogin antibody, which labels 8 of the 12 types of cone bipolar cells [13, 42] and counting cell bodies on two retinal sections from at least one mouse per timepoint (see figure legend for n values). One-way ANOVA statistical analysis was performed using GraphPad Prism software.

### Confocal microscopy

All samples were handled and chemically decontaminated according to established scrapie protocols in consultation with RML Biosafety. Samples were imaged using a Zeiss laser scanning confocal (LSM 880) microscope driven by ZEN v.2.3 software (Carl Zeiss Microscopy). A Plan Apochromat 63X/NA1.4 oil immersion lens was used, with immersion oil at a refractive index of 1.518. Image acquisition settings including laser power and gain were optimized for minimal background and cross-talk, and kept constant within an experiment for all timepoints and samples to enable direct comparisons. Stacks were collected with a lateral resolution of 43 nm and z-spacing of 130 nm except for: quantification of anti-D13 and anti-CtBP2 signals, where stacks were collected with a lateral resolution of 71 nm and z-spacing of 367 nm, with five representative fields of view acquired for each timepoint, and anti-Cone Opsin, anti-GNAT1, anti-Cone Arrestin, anti-Centrin3 which were collected with a lateral resolution/z-spacing of 18 nm/250 nm, 132 nm/250 nm, 65 nm/500 nm and 70 nm/500 nm respectively.

### Image processing and analysis

Image stacks were exported from ZEN software and deconvolved with Huygens Professional v. 20.04 (Scientific Volume Imaging, The Netherlands) using the CMLE algorithm, with SNR = 20 and a maximum of 40 iterations. The deconvolved datasets were imported to Imaris x86_64 v.9.5.1 (Bitplane AG, Zürich, Switzerland) for segmentation, surface rendering, visualization, and quantification. The average number of CtBP2 ribbons based on anti-CtBP2 signal and amount of total integrated anti-D13 signal was calculated per micron length of retina. Data were imported into Microsoft Excel for compilation, and statistical analysis was performed using GraphPad Prism v 8.3.0 (La Jolla, CA).

### Electron microscopy sample preparation

C57BL/10SnJ mice were perfused with 2% paraformaldehyde + 2% glutaraldehyde in 0.1 M Sorensen’s phosphate buffer (Electron Microscopy Sciences, Pennsylvania). Eyes were enucleated and placed in fresh fixative for at least 30 min before further for processing. The anterior portions of eyes were dissected and discarded. The remaining posterior eye cups were rinsed in phosphate buffer, followed by embedment in 2.5% low-melt agar (Precisionary, Massachusetts) made in PBS. 200 µm sections were cut with a VT1000S vibrating blade microtome (Leica Biosystems, Illinois). Sections were processed for transmission electron microscopy as follows: postfixation with 0.05% osmium tetroxide + 0.08% potassium ferrocyanide in 0.1 M phosphate buffer for 1 h, rinsed with buffer, then dehydrated in a graded ethanol series to 100%, infiltrated with LRWhite (Electron Microscopy Sciences, Pennsylvania) and polymerized overnight in homemade flat-embedding molds covered with aclar sheets at 50 °C in a vacuum oven.

For electron microscopy studies the following numbers of retinas from C57BL/10SnJ mice were taken at the given timepoints; uninfected (n = 22), 84 dpi (n = 2), 89 dpi (n = 4), 98 dpi (n = 2), 104 dpi (n = 3), 112 dpi (n = 2), 122 dpi (n = 4), 126 dpi (n = 2), 132 dpi (n = 3), 137 dpi (n = 3), 140 dpi (n = 1), 151 dpi (n = 3), 154 dpi (n = 1), 165 dpi (n = 3). These retinas were embedded in various resins (Durcupan, Araldite, HM20, and LRWhite) and examined. The LRWhite embedded retinas were selected for the imaging and comparisons shown in this paper. The numbers at each timepoint were; uninfected (n = 4), 104dpi (n = 1), 126dpi (n = 1), 132dpi (n = 1), 137dpi(n = 2), 151dpi(n = 2).

### Transmission electron microscopy

Flat-embedded vibratome sections were excised and super-glued onto resin stubs such that ultramicrotomy sections would be in the desired orientation. 70 nm sections were cut with a Ultracut UCT (Leica Biosystems, Illinois) ultramicrotome and picked up on Formvar coated 100 hex mesh copper grids (Electron Microscopy Sciences, Pennsylvania). Micrographs were acquired on a HT7800 (Hitachi, Oregon) operating at 80 kV with an XR-81B CMOS digital camera (AMT Imaging Systems, Massachusetts).

## Results

### Detection of PrPSc in retina after intracerebral scrapie injection.

**Fig. 1:**
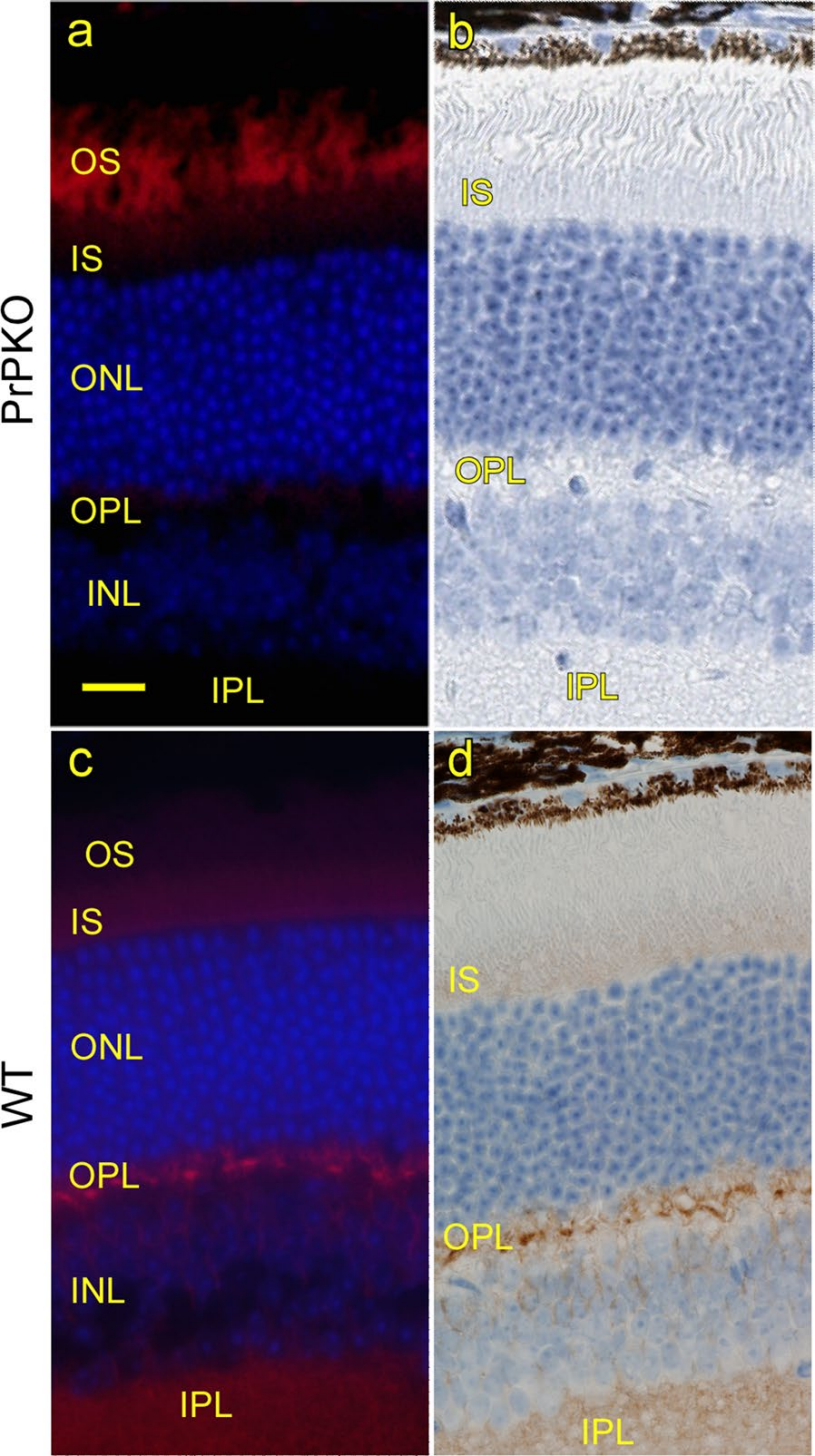
PrPC expression in uninfected PrP knockout (PrPKO) and wild-type (WT) retina using D13 antibody. **a**, **b** In PrPKO retina, normal host PrP (PrPC) was not detected by immunofluorescent stain (red) or immunohistochemical stain (brown). However, photoreceptors in OS of PrPKO retina showed red autofluorescence, a common but variable artifact of immunofluorescence studies of retina. **c**,** d** In WT retina, PrPC was observed by both staining techniques in the OPL and IPL, and weaker levels were detected in the INL and IS. Scale bar in **a** = 20 µm. *OS* outer segment, *IS* inner segment, *ONL* outer nuclear layer, *OPL* outer plexiform layer, *INL* inner nuclear layer, *IPL* inner plexiform layer

In the current experiments, prion infection of retina was achieved by intracerebral inoculation of mice with 1.0 × 10^5^ ID50 units of scrapie strain 79A. With this method, prions are known to spread to the retina via the optic tract and optic nerve [6, 16]. Progression of retinal infection was followed by immunohistochemistry (IHC) or immunofluorescence (IF) with anti-PrP monoclonal antibody D13 to detect PrP. In uninfected PrPKO mice, no PrP signal was detected by IHC or IF (Fig. 1a, b). However, in uninfected mice expressing PrP, staining of PrP was clearly detectable by IHC and IF in an irregular clumpy distribution in the outer plexiform layer (OPL) and in a more diffuse pattern in the inner plexiform layer (IPL) (Fig. 1c, d). This was similar to what has been described previously by others [10, 17, 19]. In addition, a smooth faint signal was observed in the inner segment (IS) of the photoreceptor layer (Fig. 1c, d) [54]. All of these sites were likely to represent normal cellular PrP (PrPC) as they were not seen in PrPKO mice (Fig. 1a, b).

**Fig. 2:**
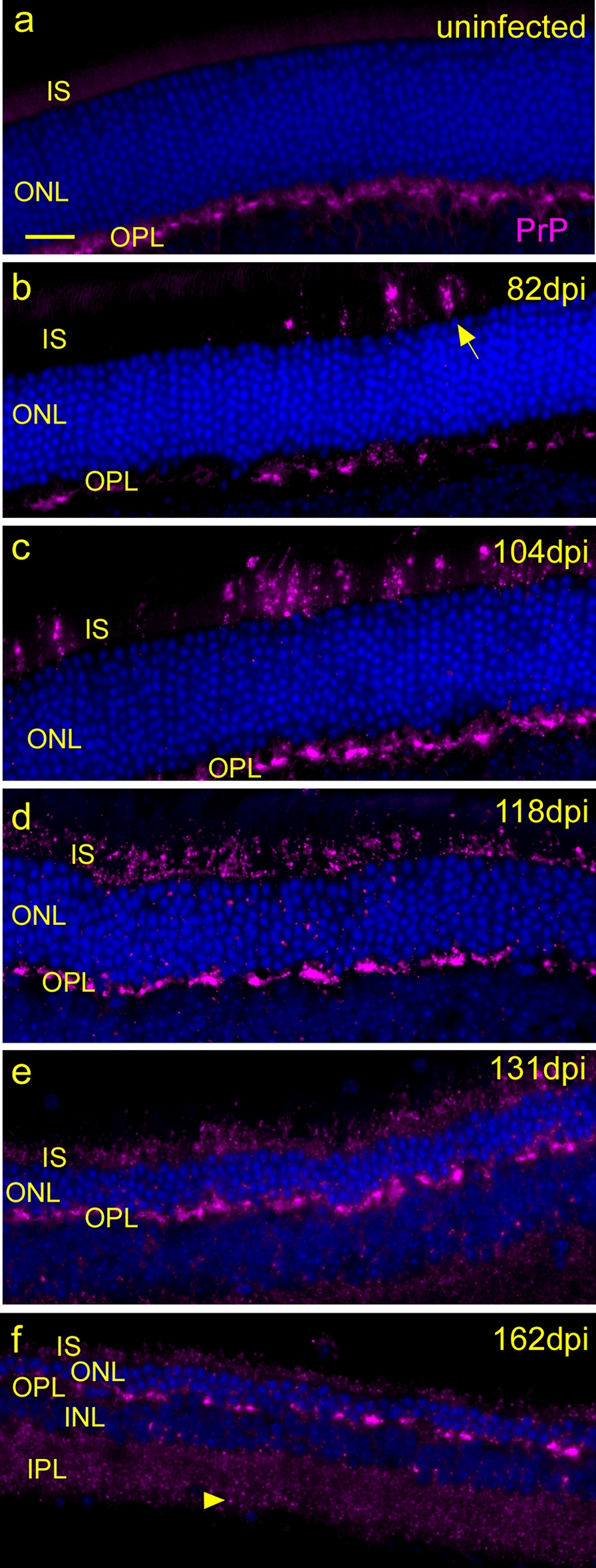
Timecourse of PrP staining with D13 antibody in retina at various times after infection with strain 79A scrapie prions. **a** Uninfected mouse showing PrPC (magenta) mainly in OPL. **b**,** c** At 82 and 104 days post infection (dpi) the misfolded, disease-associated form of PrP (PrPSc) (magenta) can now be seen in the IS and OPL at progressively wider areas. **d** At 118 dpi, PrPSc is widespread in the IS and deposits are not restricted to discrete individual cells. Some small deposits are visible in the ONL and ONL is beginning to thin as photoreceptors die. **e** At 131 dpi, PrPSc is deposited in the IS and OPL, and ONL is much thinner. **f** PrPSc staining appears less at 162 dpi in IS and OPL, and ONL is dramatically thinner. Punctate PrPSc is present in IPL (arrowhead). Scale bar in **a** = 20 µm

At 82 days post-infection (dpi), bright punctate PrP staining in patchy aggregates of varying sizes were seen in an irregular scattered distribution along the IS and OPL layers (Fig. 2b). This staining was likely to be disease-associated PrPSc, as it was not seen in uninfected mice (Figs. 1c, d, 2a). At 104 and 118 dpi, the PrPSc staining in the IS and OPL was more extensive, and at 118 dpi, PrPSc staining was often seen on the entire circumference of the IS and OPL (Fig. 2c, d). At 118 dpi, there was also a decrease in the width of the outer nuclear layer (ONL) indicating a loss of photoreceptor cell nuclei. At 131 dpi, the ONL was further reduced in size, and the PrPSc staining in the IS and OPL was decreased in intensity (Fig. 2e). At 162 dpi, all retinal layers were thinned (Fig. 2f), and the ONL was now only 2–3 cells thick in most areas. Faint PrPSc staining was further decreased in the IS and OPL regions but was now slightly more detectable in the inner plexiform layer (IPL). Interestingly, this type of neurodegeneration was not seen in uninfected PrPKO mice, which suggested that loss of PrP does not itself cause photoreceptor degeneration (unpublished results from our group).

The time course of PrPSc deposition and retinal degeneration seen in these experiments using indirect IF was similar to our previous data using detection of PrP by IHC [54]. However, the details of the PrPSc aggregate morphology was seen more clearly by IF. Since photoreceptor rods and cones are the main cells normally present in the IS region and these cells also have processes extending into the OPL, early PrPSc deposition was likely to be associated with one or both of these photoreceptor cell types. To study the cell types associated with PrPSc in this model, we used the IF method with dual staining for PrP and antibodies reactive with cone- or rod-specific proteins (Tables 1, 2).

### Deposition of PrPSc occurs first in cone photoreceptors

**Fig. 3:**
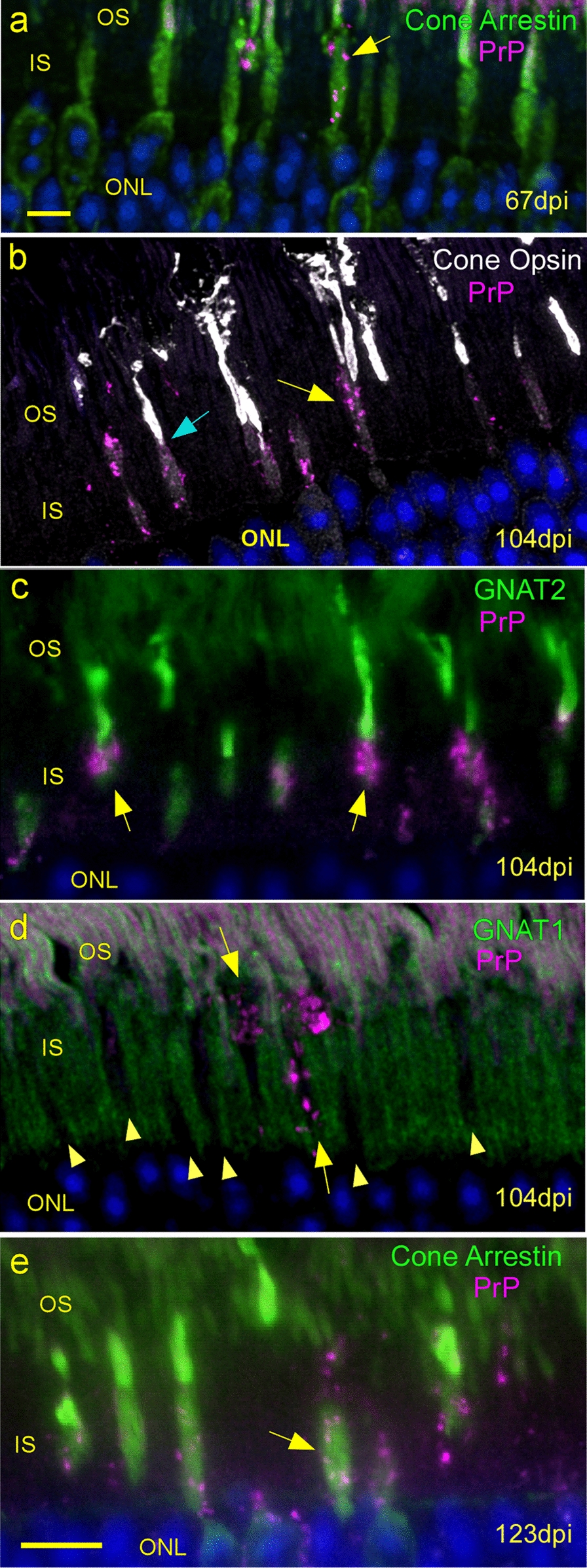
PrPSc accumulates first in cone photoreceptor inner segments.** a** At 67 dpi, rare small punctate PrPSc deposits (arrow) are present on cone photoreceptors marked with Cone Arrestin (green). Magenta in outer segment is autofluorescence of rhodopsin. **b** Cone opsin (white) marks cone outer segments with PrPSc (magenta) deposits associated mainly with cone inner segments (yellow arrow) at 104 dpi. The transition from faint to intense cone opsin staining marks the boundary between cone IS and cone OS (blue arrow). **c** Another cone-specific outer segment protein GNAT2 (green) shows obvious connection with PrPSc (magenta) staining cone inner segments at 104 dpi (arrows). **d** At 104 dpi, rod-specific marker, GNAT1 (green) stains rod inner and outer segments, but spares cones (arrowheads). PrPSc (magenta) accumulations are present in the dark GNAT1-free areas (arrows). **e** A separate experiment done with 22L scrapie strain shows same association of PrPSc with cone inner segments at 123 dpi, suggesting cone specificity is not strain-specific. Scale bar = 5 µm. **a**,** b**,** d** are confocal z-stacks, **c**,** e** are widefield images

At 67 dpi, PrPSc was detected in a few individual cells in the IS region, and this PrPSc was associated with detection of cone arrestin in the same individual cells (Fig. 3a). Similarly, at 104 dpi, PrPSc staining was associated with detection of cone opsin (Fig. 3b) and GNAT2 (transducin alpha-2), another cone-specific marker (Fig. 3c). In both of these examples, the PrPSc staining was in the inner portion of the IS, whereas both the cone opsin and GNAT2 proteins were mostly in the outer segment (OS), i.e. distal to the PrPSc but appearing to be in the same individual cells as the cone-specific marker proteins. In contrast, GNAT1 (transducin alpha-1), a rod-specific protein, did not co-associate with PrPSc at 104 dpi which was located in the dark spaces not stained by GNAT1, i.e. cones (Fig. 3d). Because scrapie strains have been shown to show cell-specific infectivity [8], we tested the 22L strain, which also targeted cone photoreceptors before rods (Fig. 3e). These observations indicated that PrPSc deposition appeared first in cone photoreceptor inner segments. Additional studies showed that rods were also infected starting around 118 dpi (see below).

**Fig. 4:**
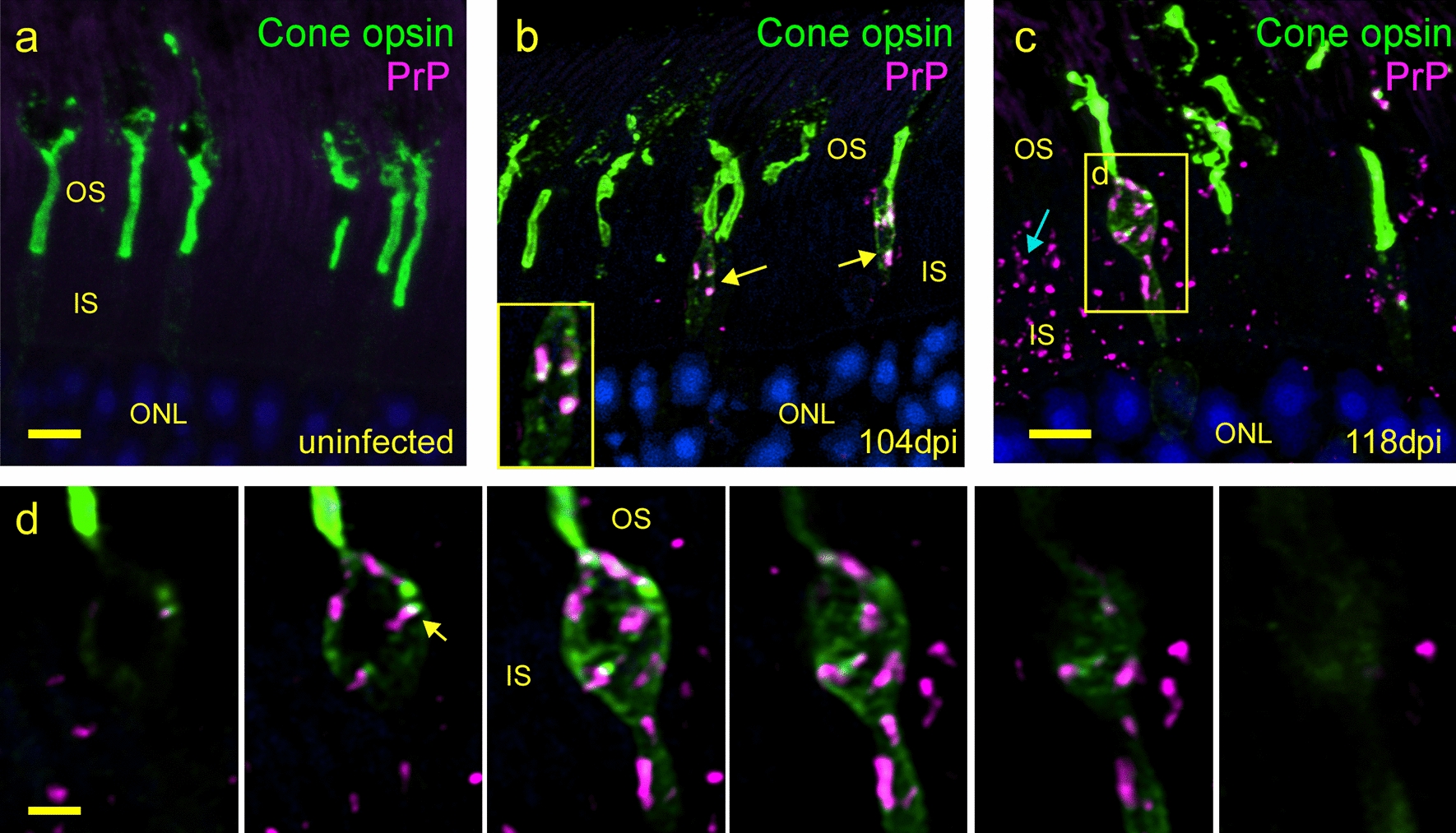
Early PrPSc accumulation associated with cone photoreceptor damage.** a** Uninfected retina shows normal cone morphology and distribution of cone opsin (green) in outer segment.** b** At 104 dpi early PrPSc deposits are associated with cone inner segments (arrow) and some PrPSc appears to localize with cone opsin (white areas in × 2 magnified inset). **c** At 118 dpi, a swollen, dystrophic cone with PrPSc is seen (yellow box) and some PrPSc is also present in rods (blue arrow). **d** Serial confocal sections spaced 0.5 µm apart, magnify the swollen cone inner segment from **c** and suggests the PrPSc is inside the inner segment along with mistrafficked cone opsin. Scale bar in **a** and **c** = 5 µm. Scale bar in **d** series = 2 µm. **a–c** are confocal z-stacks

Retinas were next studied by confocal microscopy to look for cone damage by dual staining with anti-PrP and anti-cone opsin. At 104 dpi, swelling in the inner segment portions of some PrPSc-positive cones was observed (Fig. 4b). This swelling was never seen in uninfected retinas (Fig. 4a), and therefore appeared to be a sign of damage due to prion infection. This swelling was typical of necrotic cell death as described in cone degeneration in a rd10 mouse model [37]. At 118 dpi abundant PrPSc was seen in cones and in the inner segment cells lacking cone opsin (Fig. 4c), indicating that prion infection had spread to rods by this time. Six serial optical sections through the cone outlined in Fig. 4c revealed that PrPSc (red) and cone opsin (green) were mostly separated inside this cone (Fig. 4d). However, the detection of cone opsin outlining the outer edge of the cytoplasm was not seen in cells lacking PrPSc, suggesting that this unusual distribution of cone opsin may be a manifestation of prion-induced damage in this cone.

### Detection of PrPSc-associated damage in the inner segment

**Fig. 5:**
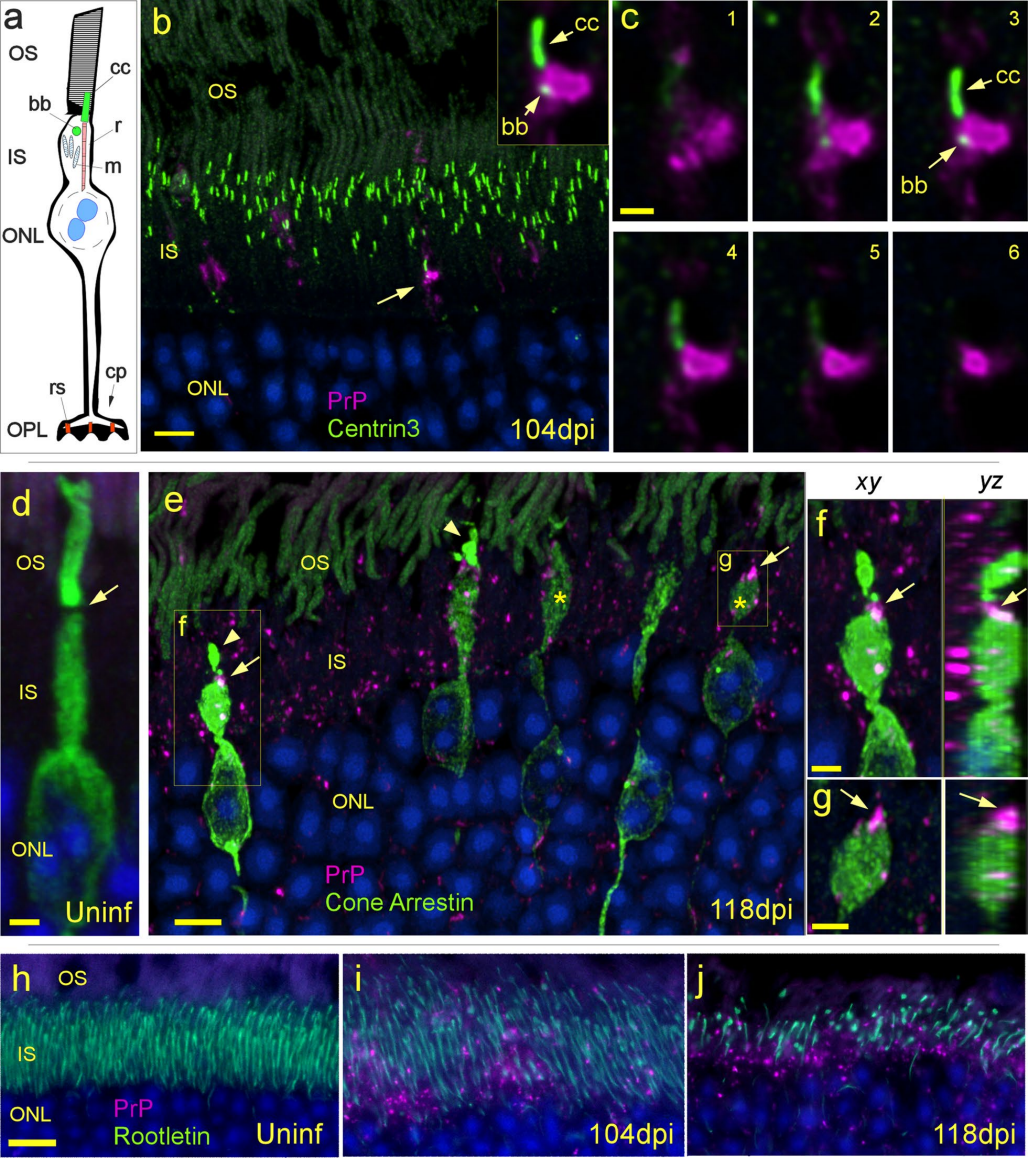
Early PrPSc accumulation and damage in the inner segment. **a** Cartoon of cone photoreceptor shows key structures related to PrPSc deposition. *cc* connecting cilium, *bb* basal body, *r* rootlet, *m* mitochondria, *cp* cone pedicle, *rs* ribbon synapse. **b** Anti-centrin3 antibody (green) marks the connecting cilium and basal body (small green dots) of all photoreceptors. PrPSc (magenta) accumulation at entrance to connecting cilium (arrow), magnified in inset. **c** Serial 0.5 µm confocal sections (**c1–6**) showing relative localization of PrPSc, cilium and basal body. **d** Cone arrestin (green) staining of uninfected cone photoreceptor. Arrow indicates likely position of connecting cilium. **e** 118 dpi shows retina stained for cone arrestin (green) and PrP (magenta). Asterisks mark cones missing outer segments, yellow arrows point to position of connecting cilia and associated PrPSc (magenta) deposit, and arrowheads show dystrophic outer segments. **f**,** g** Confocal analysis showing xy and yz planes of swollen cones from **e** confirm the presence of PrPSc at the location of the connecting cilium (arrow) between the IS and OS. **h** In an uninfected retina**,** anti-rootletin (green) antibody stains the rootlets of photoreceptors. **i**, **j** At later days post infection, PrPSc (magenta) is increased, rootlets are fewer in number and misshapen. Scale bars: **b**,** e** = 5 µm; **c** = 1 µm; **d** = 2 µm; **f**,** g** = 2 µm; **h** = 10 µm. **b**,** d**,** e** Maximum intensity projections (MIP) of confocal z-stacks **h**, **i**, **j** are widefield images

To understand how PrPSc deposition might be causing damage to cone (and rod) photoreceptors we looked for association of PrPSc with additional key inner segment structures. First, the relationship between PrPSc deposition and the cilium connecting the inner and the outer segments of the photoreceptor cells (Fig. 5a) was examined by dual staining for PrP and Centrin3, a protein located within the cilia of rods and cones. Early in disease, at 104 dpi, deposition of PrPSc could often be detected near the entry point of the connecting cilium (Fig. 5b). In some cases, PrPSc was directly adjacent to the cilium, as confocal analysis showed that PrPSc, basal body and cilium could be found within the same section (Fig. 5c). The deposition of PrPSc at this site may have affected the cilium’s ability to transport phototransduction proteins between the IS and OS. Ciliary dysfunction is known to cause photoreceptor death in other forms of retinal degeneration [43].

**Fig. 6:**
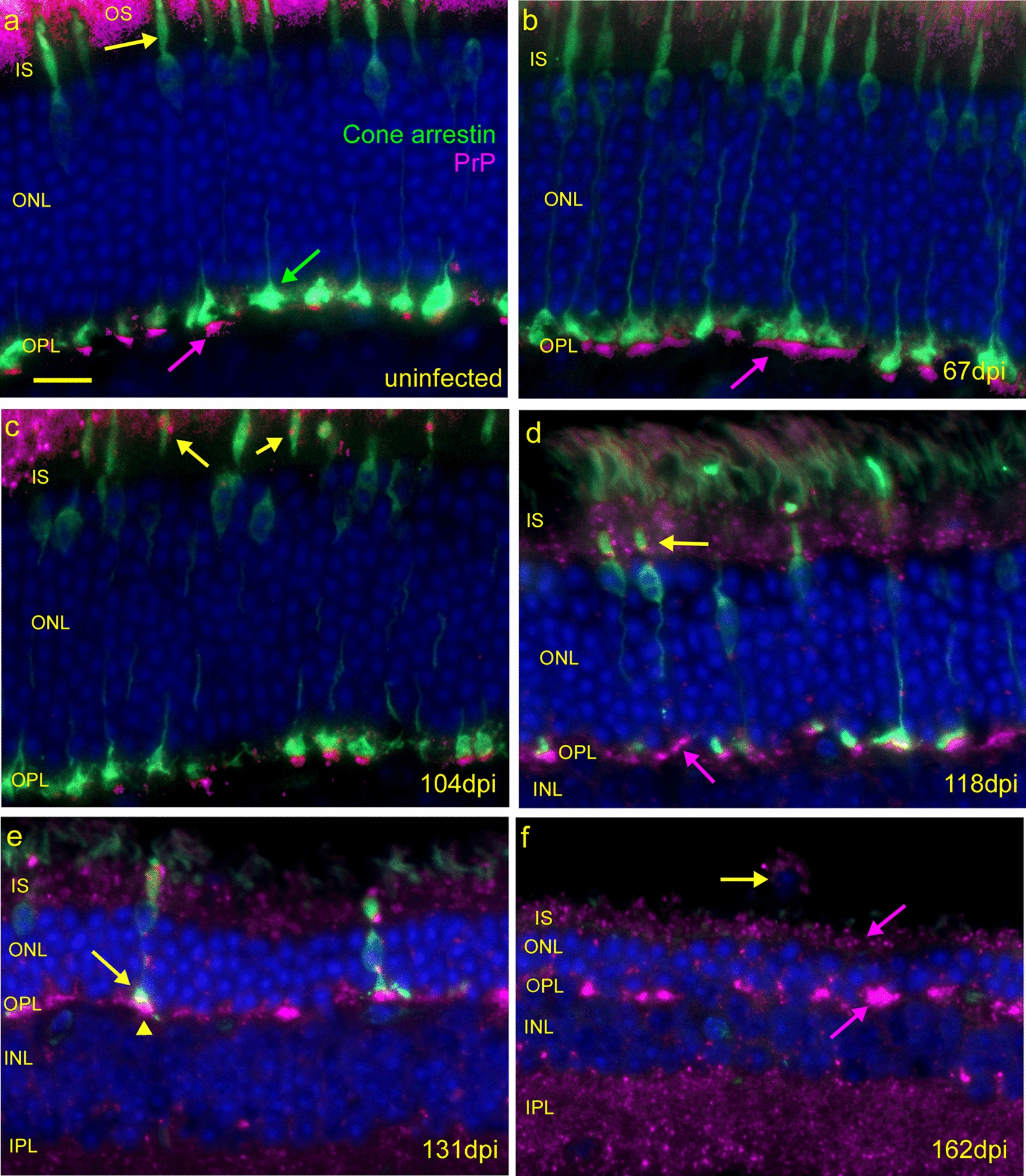
Timecourse of damage to cone photoreceptors. **a** Anti-Cone arrestin (green) stains cone photoreceptor, inner segments (yellow arrow) and pedicles (green arrow) in an uninfected mouse. Anti-PrP antibody D13, stains PrPC (magenta) at the base of cone pedicles in the OPL (purple arrow). Autofluorescence is also present in the OS. **b** At 67 dpi cone arrestin and PrP are distributed similarly to uninfected retina (magenta arrow). **c** Small deposits of PrPSc are present in the IS at 104 dpi and are associated with cones (arrows). **d** At 118 dpi, the number of cones is reduced (green), most cone pedicles have disappeared from the OPL and the remaining cones are dystrophic (yellow arrow), missing their outer segments. PrPSc is widespread in the IS and ONL. **e** Few cones (green) remain at 131 dpi, pedicles (arrow) are much smaller in size and associated with dense clumps of PrPSc (arrowhead). **f** Cones are not detectable at 162 dpi, and ONL is 2–3 nuclei thick, suggesting rod photoreceptors have mostly died. A macrophage or microglial cell (yellow arrow) containing PrPSc is present in the remnants of the OS. Scale bar in **a** = 20 µm, applicable to all images

To test this idea, we stained for cone arrestin, a cone-specific phototransduction protein, known to be trafficked between the IS and OS [2, 49]. In uninfected retinas, cone arrestin was present in the OS, IS, ONL (Fig. 5d) and pedicles (Fig. 6). The cone outer segments of a 118 dpi retina appeared shrunken and malformed compared to an uninfected cone and in some cases, outer segments were absent (Fig. 5d–g). Inner segments of cones in the 118 dpi retina were often swollen, and PrPSc deposits could be found at the constriction point between the IS and OS, i.e. the location of the connecting cilium (Fig. 5d–g). Together these data suggest that deposition of PrPSc in the inner segment may have affected the ciliary transport of cone arrestin and/or other proteins. We also investigated the association of PrPSc deposits with another prominent inner segment structure, the rootlet, which functions to stabilize the connecting cilium [59] (Fig. 5a). For this, we used an anti-rootletin antibody together with D13 anti-PrP. As PrPSc appeared to accumulate in the inner segment at 104 and 118 dpi, rootlet morphology changed, and the density of rootlets decreased (Fig. 5h–j). While PrPSc was not usually associated with rootletin, these data suggested that PrPSc accumulation may have damaged the inner segment and its structures including the rootlets.

### Detection of PrPSc-associated damage in the outer plexiform layer

The early accumulation of PrPSc and damage to cone inner segments led us to examine cone photoreceptors more closely. We used dual staining with anti-PrP plus anti-cone arrestin to follow the progression of PrPSc deposition and changes in overall cone morphology over the time course of prion infection from 67 to 162 dpi.

In uninfected retina, small dense patches of PrP staining were seen in the OPL just vitread (toward the vitreous) to many of the cone pedicles expressing cone arrestin (Fig. 6a), and this was assumed to be PrPC as it was seen in uninfected mice. Similar staining was also seen at 67 dpi (Fig. 6b). At 104 dpi, PrP staining in the OPL was seen basal to some of the cone pedicles similar to uninfected mice. More obvious changes were seen at 118 dpi, where cone pedicles were fewer in number and smaller in size (Fig. 6d). PrPSc was abundant in the IS region on both cones and rods, and PrPSc staining in the OPL was both punctate and diffuse and appeared to be independent of cone pedicles (Fig. 6d). At 131 and 162 dpi, PrPSc staining in IS and OPL remained strong and was similar to 118 dpi. However, at these times, most cone pedicles were gone, and the ONL showed thinning due to a large loss in both cone and rod cell nuclei (Fig. 6e, f). These data suggested that PrPSc deposition in the IS and OPL regions might have induced pathogenic effects resulting in death of both cones and rods.

### Loss of cone pedicles and rod spherules visualized by GLUT1 staining

Glucose transporter 1 (GLUT1) is known to be an excellent marker for cell surface visualization in tissues. Therefore, we used anti-GLUT1 and anti-PrP dual staining to study the details of PrP localization relative to cone pedicles and rod spherules in the OPL area following prion retinal infection. Prior to prion infection, cone pedicles were detected as triangular dark shaped spaces outlined by GLUT1, and just vitread to many of these pedicles a patch of clustered coarse PrPC staining was observed (Supp Fig. 1a). In addition, in the same figure, numerous rod cell endings, i.e. spherules, were also seen, and these appeared to be smaller rounded dark shapes outlined by GLUT1 mainly sclerad (toward the sclera) to cone pedicles (Additional file 1: Fig. 1a). Following prion infection at 118 dpi, cone pedicles were difficult to recognize or had disappeared and rod spherules were decreased in number and size (Additional file 1: Fig. 1b). Later, at 153 dpi, most of the spherules and pedicles were missing, and all the photoreceptor layers (OS, IS, ONL, OPL) were degenerated (Additional file 1: Fig. 1c). These results demonstrated progressive damage to both rods and cones associated with the presence of PrPSc deposition sclerad to and within the OPL.

### Comparative detection of PrP, ribbon synapses and other structures in uninfected retina

**Fig. 7:**
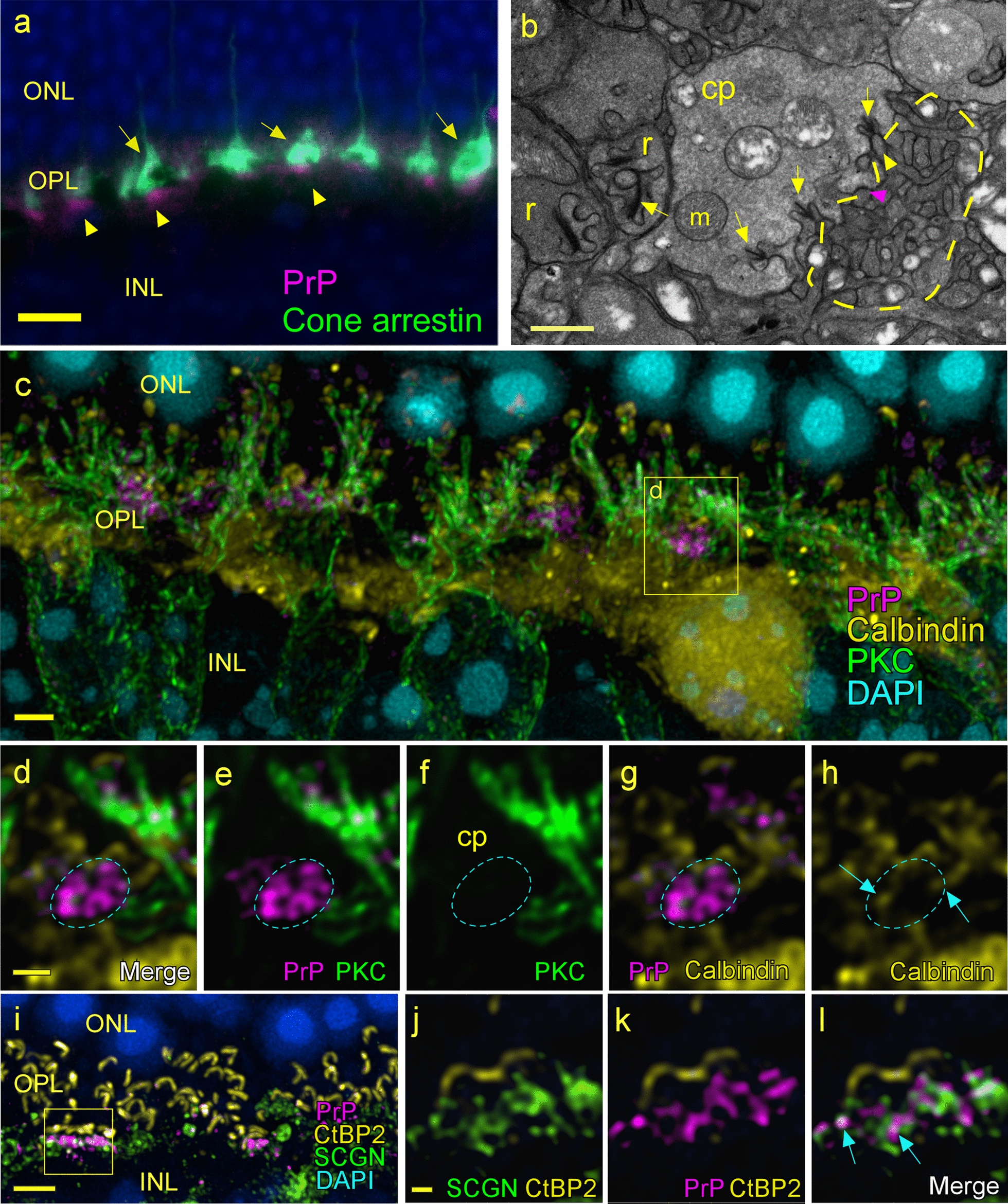
In uninfected retina, PrPC expression is concentrated at the base of cone pedicles. **a** In the OPL, PrPC (yellow arrowheads) staining is present at the base of cone pedicles highlighted with anti-cone arrestin (arrows). **b** Electron micrograph (TEM) of the OPL showing a cone pedicle (cp), with multiple round mitochondria (m) and three ribbon synapses (arrows). A yellow dotted line surrounds an estimated region of PrPC from **a**, which contains many dendritic processes from bipolar and horizontal cells. Some dendrites synapse at ribbons (yellow arrowheads) or make flat contact type synapses (magenta arrowhead) with cone pedicles. Ribbon synapses (arrows) are also visible in rod spherules (r). **c** Confocal z-stack of uninfected retina shows gross relationship of PrP to rod bipolar cells (PKCα) and horizontal cells (Calbindin). **d–h** A high magnification single 0.130 µm optical section taken from area in box in panel **c**, shows PrP is not associated with PKCα and that some calbindin-positive processes (arrow) lie within the PrP-positive area, but association is not clear. **i** Confocal z-stack image of retina stained for cone bipolar cell dendrites (SCGN), ribbons (CtBP2) and PrP, shows a dense patch of PrPC vitread to a cluster of short ribbons (box) in a cone pedicle. **j–l** A single 0.130 µm optical section taken from within the box in panel e shows PrPC in close association with SCGN-positive dendrites, and some colocalization is seen as white (arrows). Scale bar in **a** = 10 µm, **b** = 1 µm, c = 2 µm, d = 1 µm, i = 4 µm, j = 0.5 µm

In the photoreceptor layer bordering the OPL, cone and rod photoreceptor cells are connected with processes from horizontal cells and rod or cone bipolar cells to form ribbon synapses, which have unique electrophysiological and morphological features [36]. Initially we compared the locations of several of these structures in uninfected mice to determine their normal morphology, as observed by both confocal and electron microscopy. Dual staining of an uninfected mouse for cone arrestin and PrP showed PrPC located in large dense clusters vitread to cone pedicles (Fig. 7a). Electron microscopy of the OPL demonstrated rod spherules with single large ribbon synapses and cone pedicles containing multiple short ribbon synapses (Fig. 7b). Notably, comparisons of EM and light microscopy images suggested that areas of intertwined dendrites from bipolar and horizontal cells (Fig. 7b) corresponded to the clustered areas of PrPC seen in Fig. 7a.

In order to understand the association of PrPC with these structures, uninfected retina was stained simultaneously with antibodies against PrP and proteins expressed by horizontal and bipolar cells (Fig. 7c–f). Confocal microscopy analysis of full z-stacks and individual 130 nm optical sections was used to reveal the relative locations of the processes and PrP. Dendrites of rod bipolar cells were marked with anti-PKCα and although they were present very near the PrP-stained area beneath cone pedicles, they were not directly associated with PrP (Fig. 7c, d). Likewise, Calbindin-positive horizontal cell processes were not clearly associated with the dense clusters of PrP (Fig. 7c–h). Next, we triple-stained with anti-PrP, anti-CtBP2, a marker for ribbons and anti-Secretagogin, a protein expressed in 8 of the 12 types of cone bipolar cells [13, 42]. In this experiment, dendrites of cone bipolar cells expressing secretagogin (SCGN) showed a close association with PrPC (Fig. 7i–l). These results suggested that PrPC is highly expressed very near cone pedicles and thus may explain the early accumulation of PrPSc in cones versus rods.

### Damage to ribbon synapses seen by staining with anti-CtBP2

**Fig. 8:**
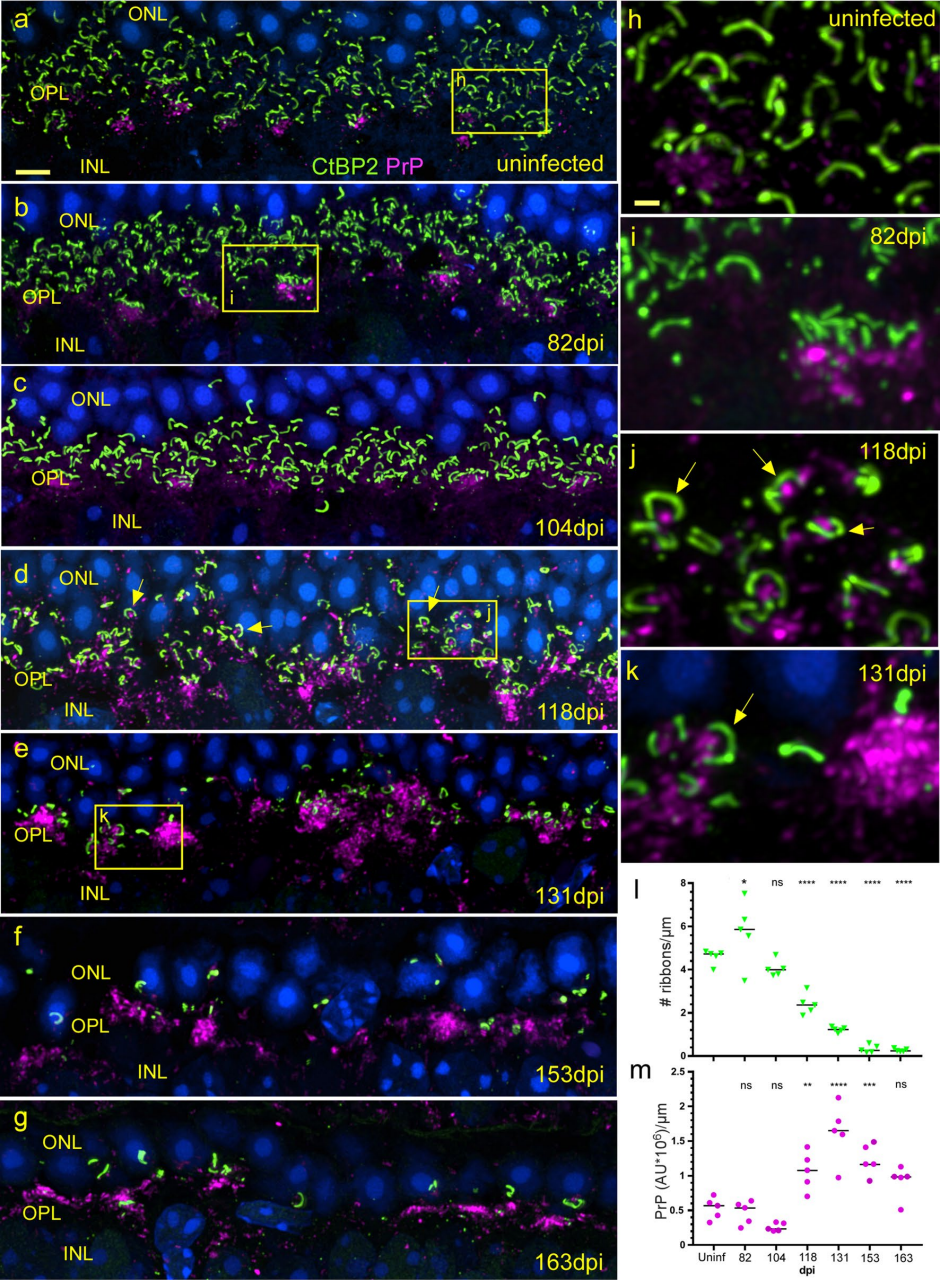
Timecourse of disappearance of ribbon synapses in OPL. **a**, **h** Uninfected retina shows many horseshoe-shaped ribbons stained with CtBP2 (green), sclerad to PrPC (magenta) densities. Most ribbons are not associated with PrPC see panel h. **b**, **i** At 82 dpi, the number of ribbons is similar to uninfected, but at cone pedicles PrP appears brighter and more punctate suggestive of new PrPSc accumulation. **c** 104 dpi retina appears similar to 82 dpi. **d**, **j** 118 dpi, ribbons in OPL appear disorganized and are significantly reduced in number, and PrPSc deposits are visible within horseshoe arcs, but not touching, most ribbons (**j**, yellow arrows). **e**,** k** At 131 dpi PrPSc accumulation has peaked and most ribbons have disappeared**.** Remaining horseshoe-shaped ribbons show association with PrPSc (**k**, yellow arrow). **f**,** g** At late dpi, ONL has thinned dramatically, few ribbons remain, PrPSc is widespread. **l** Graph shows # of ribbons/µm of OPL of scrapie infected retina at dpi vs uninfected. Each dot represents count from one field of view. **m** Quantitation of PrP (magenta) fluorescence integrated intensity/µm of OPL of scrapie infected retina at dpi vs uninfected. Each dot represents integrated intensity measurements from one field of view. *Lines* median value, *ns* not significant, *p < 0.05, **p < 0.01, ***p < 0.001, ****p < 00.0001. Statistics were calculated using one-way ANOVA with multiple comparisons. Scale bars: **a–g** = 4 µm; **h–k** = 1 µm

Using dual staining immunofluorescence combining anti-PrP with anti-CtBP2 antibodies specific for ribbon structures, we studied the development of PrPSc-associated alterations on ribbon synapse components over time after prion infection. In both uninfected and 82 dpi retinas, ribbon synapses were seen sclerad to the OPL as a continuous band of linear or horseshoe-shaped structures, and PrP was mostly in bunched loose aggregates vitread to the lower-most ribbons (Fig. 8a, b). At 104 dpi, ribbons appeared to be similar to 82 dpi, but PrP staining was slightly more prominent in the ribbon area than previously (Fig. 8c). At 118 dpi (Fig. 8d), ribbons were significantly decreased in number (Fig. 8l), and PrP was deposited among the ribbons rather than below them (Fig. 8d, j) compared to uninfected retina and earlier timepoints. This PrP was very likely PrPSc as staining was not seen in this location in uninfected retina. Interestingly, at higher magnification, small accumulations of PrPSc were detected within the horseshoe-like curves of many of the ribbons (Fig. 8j, k). Moreover, PrPSc accumulations were never found directly on ribbons, which is where synaptic vesicles are located. This suggested that PrPSc was on the postsynaptic side of the ribbon synapses (Fig. 8j). At later time points, 131, 153 and 163 dpi, ribbons were progressively destroyed, as were the cone and rod cell nuclei in the ONL (Fig. 8e–g, l). Disappearance of ribbons correlated with significant increases of PrPSc at 118 dpi (Fig. 8l vs m). Thus, the appearance of PrP within the ribbon horseshoes correlated with the onset of loss of ribbon synapses and suggested this PrPSc deposition might be responsible for this damage.

### Association of PrPSc to rod and cone bipolar cells near ribbon synapses

The location of PrPSc, adjacent to but not touching ribbons, suggested these deposits were associated with the postsynaptic portion of the ribbon synapses. Dendrites of rod and cone bipolar cells and horizontal cells are known to make up the postsynaptic elements of ribbon synapses in the OPL [36]. Here, investigation of the possible interactions of PrPSc with rod bipolar cells was done by dual staining with anti-PrP and anti-PKCα, which is specific for rod bipolar cells.

**Fig. 9:**
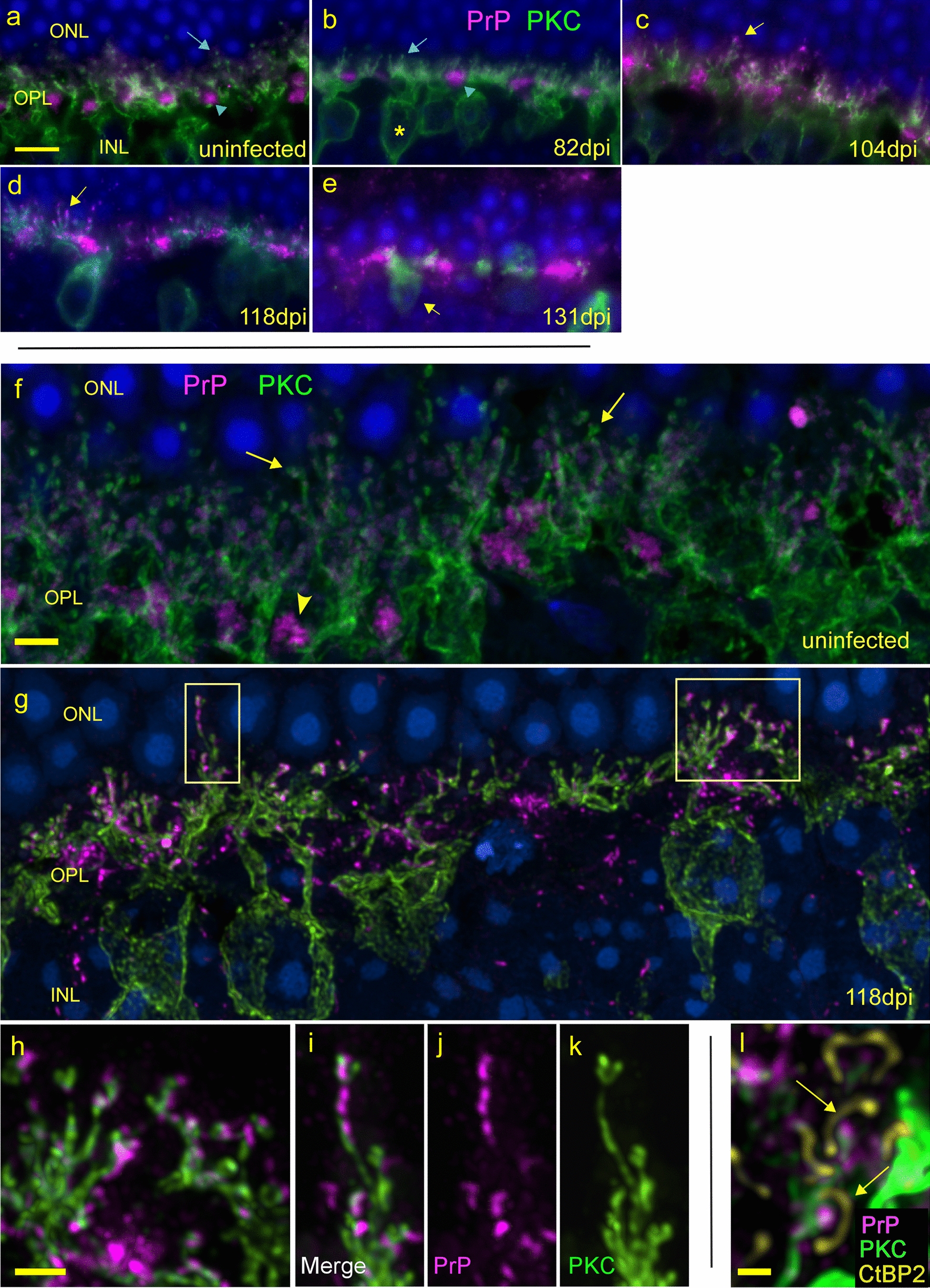
PrPSc accumulation at tips of rod bipolar cell dendrites and disappearance of dendrites. Epifluorescent microscopy analysis. **a** In uninfected retina, PrP (magenta) (blue arrowhead) and PKCα-positive rod bipolar cells (green) are seen near each other in the OPL. Many boutons (blue arrow) are present at dendritic tips of rod bipolar cells. **b** At 82 dpi, OPL appears similar to uninfected. Asterisk marks a rod bipolar cell body in INL. **c** At 104 dpi, PrP staining appears at dendritic tips. **d** At 118 dpi, most PKCα-positive dendrites display PrP at tips of processes (boutons) (arrow). **e** By 131 dpi most dendrites and dendritic boutons have disappeared, bipolar cell bodies remain (arrow), and PrPSc is distributed in large aggregates. Confocal analysis. **f** Maximum intensity projection shows PKCα and PrP staining of uninfected retina. Arrows indicate PKCα-positive rod bipolar cells with obvious boutons at the ends of dendrites. **g** A 118 dpi retina shows PrPSc arranged in bead-like deposits on dendrites, often replacing boutons. Magnification of boxes on left and right are shown below in panels **h**–**k**. **h** Confocal image shows association of PrPSc with dendrites and dendritic tips in area of right box from **g**. **i–k** Confocal images show merged, PrP and PKC staining of left box area in **g**. **l** In a high magnification of confocal image, anti-CtBP2 antibody marks ribbons (arrows) and confirms PrPSc deposits (magenta) on tips of dendrites (green) invaginating, but not touching, ribbons. Scale bars: **a–e** = 5 µm; **f**, **g** = 3 µm; **h–k** = 2 µm; **l** = 0.5 µm

In uninfected and 82 dpi prion-infected mice, cell bodies of rod bipolar cells were seen just vitread to the OPL and dendritic processes from these cells extended up through the OPL to the area of the cone pedicles and rod spherules (Fig. 9a, b). PrP was detected mostly in the OPL sclerad to the rod bipolar cells (Fig. 9a, b). However, starting at 104 dpi and continuing at 118 dpi, PrP deposits were seen in a new location, at the tips of the dendritic processes of the rod bipolar cells that synapse with ribbons (Fig. 9c, d). This was also visible in more detail by confocal microscopy (Compare Fig. 9f vs g). Because deposits in these locations were only seen after prion infection, they were thought to be PrPSc.

**Fig. 10:**
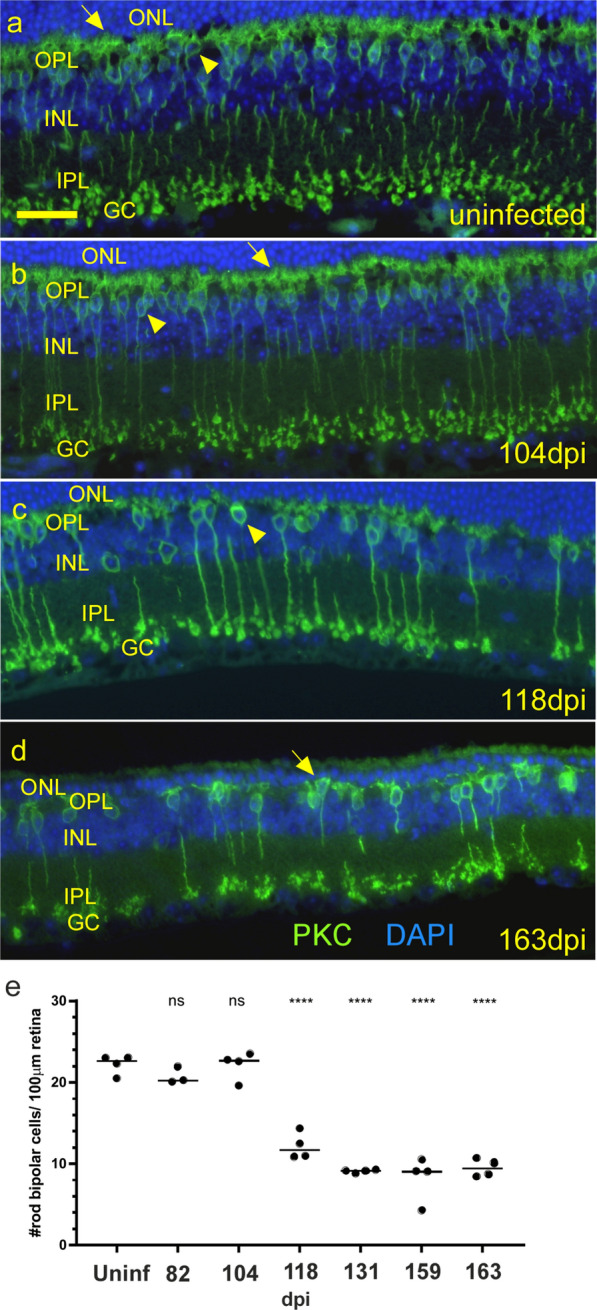
Changes in rod bipolar cells during infection. **a** anti-PKCα staining (green) in an uninfected mouse shows normal distribution and morphology of rod bipolar cells. Numerous dendrites (arrow) reach upward to synapse with photoreceptors in ONL from each cell body (arrowhead). **b** At 104 dpi rod bipolar cells appear similar to uninfected mouse. **c** By 118 dpi the number of bipolar cells has dropped to about half vs uninfected and some cell bodies have few if any associated dendrites (arrowhead). **d** Late in disease, at 163 dpi, rod bipolar cell number is significantly reduced compared to uninfected retina, and few dendrites reach into OPL (arrow). **e** The number of rod bipolar cells per 100 µm of retina over time. Each dot represents counts from one field of view, line is median, *ns* = not significant, *p < 0.05, **p < 0.01, ***p < 0.001, ****p 0.0001 statistics by one-way ANOVA with multiple comparisons test. Scale bar in **a** = 20 µm

At higher magnification this PrPSc had the appearance of beads on a string extending along the processes (Fig. 9h–k), and PrPSc seemed to displace or overlap the original PKCα staining of the processes. This was specific for infected retinas at the 104–118 dpi time-points (Fig. 9c, d, g) and was not seen in the uninfected or 82 dpi retinas (Fig. 9a, b). The precise location of PrPSc deposits, at the ribbon synapse on the tips of rod bipolar cell dendrites, was confirmed using confocal analysis of a section triple stained for PrP, PKCα and CtBP2 (Fig. 9l). At late times in disease (131 dpi), the majority of the rod bipolar dendritic structures had degenerated (Fig. 9e), however, rod bipolar cell bodies appeared to be decreased only by about 50% starting at 118 dpi and they did not decrease further after this time (Fig. 10a–e). Axons, dendrites and dendritic boutons disappeared along with the cell bodies. This was in marked contrast to the rods and cones themselves which were mostly eliminated at this time post-infection.

**Fig. 11:**
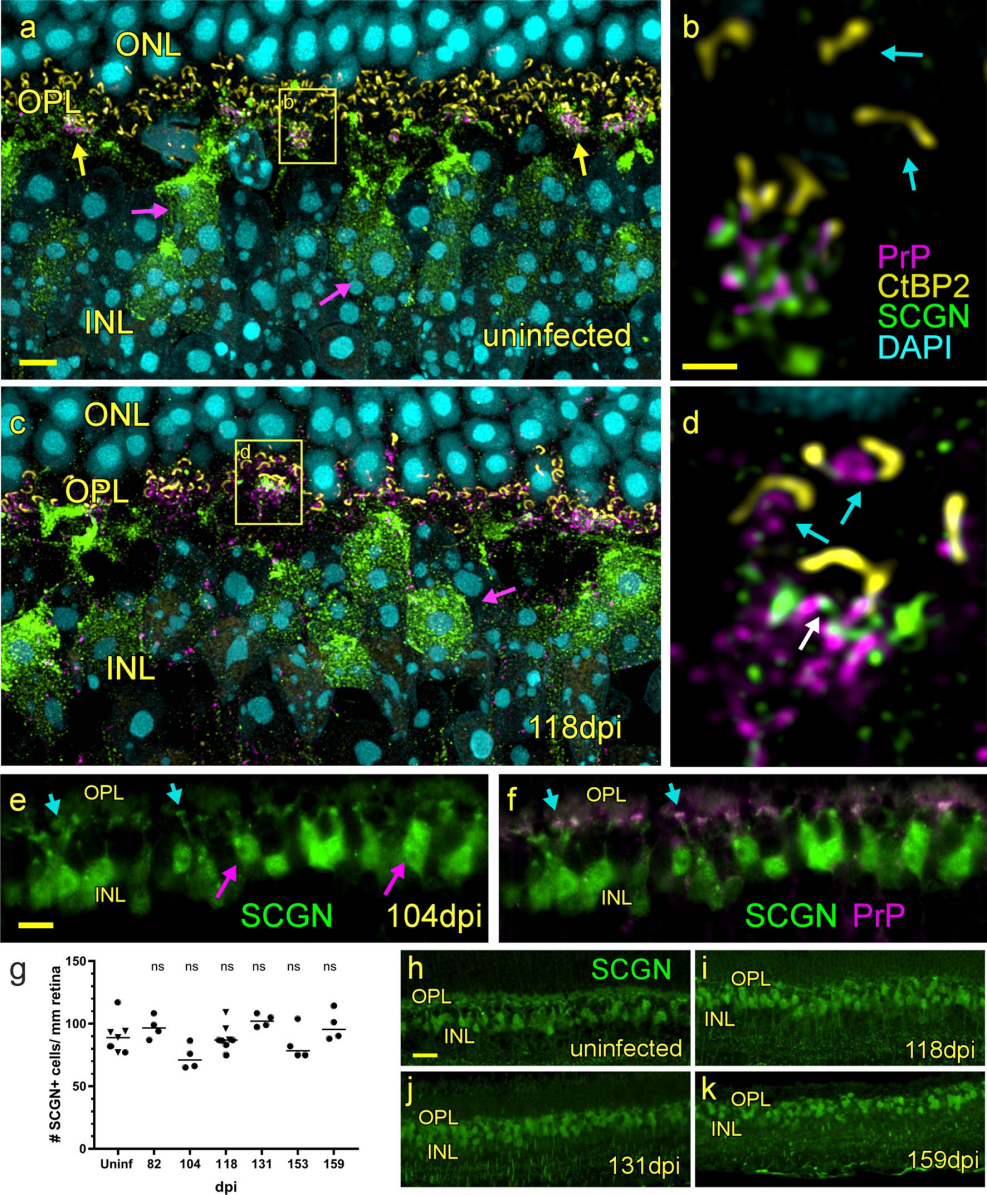
Association of PrPSc with cone bipolar cells. **a** Confocal z-stack of an uninfected retina shows secretagogin-positive cone bipolar cell bodies (magenta arrows) and their green dendritic processes associated with PrP (magenta) in dense clusters (yellow arrows) which include short ribbon synapses (yellow). **b** A single confocal section taken from the box in panel** a** shows PrP in close association with cone bipolar cell dendrites (green), beneath clusters of short ribbons (yellow) typically found in cone pedicles. Horseshoe-shaped ribbons in rod spherules (blue arrows) are not associated with PrP or SCGN. **c** In a confocal z-stack, at 118 dpi, cone bipolar cell bodies (magenta arrow) appear normal but PrP staining is increased in the OPL. **d** A single confocal section taken from the box in panel c shows increase in PrP among the SCGN-positive dendrites with some PrP overlapping SCGN, suggesting colocalization (white arrow). PrP is also present within the horseshoe-shaped ribbon synapses in rods (blue arrows). **e**, **f** Widefield images at 104 dpi show numerous examples of PrP clusters localizing with the ends of SCGN-positive dendrites (blue arrows) originating from cone bipolar cells (magenta arrows). **g** Number of cone bipolar cells per mm vs dpi. Each symbol represents the count from one field of view. Different symbols represent different animals**.** Line represents median. **h–k** anti-Secretagogin stained retina from uninfected and three different dpi show relative numbers of SCGN-positive cells. ns = not significant, statistics by one-way ANOVA with multiple comparisons test. Scale bars: **a**, **c** = 4 µm; **b, d** = 1 µm; **e,f** = 10 µm; **h–k** = 25 µm

The dendrites of *cone* bipolar cells synapse mainly with cone photoreceptor cells in the OPL and can be marked using anti-Secretagogin (SCGN) antibody [13]. To check for an association of PrPSc deposits with cone bipolar cell dendrites we triple-stained retina for PrP, SCGN and CtBP2 (Fig. 11a–d). As was also shown in Fig. 7, PrPC in uninfected mice was found to be in close association with SCGN-positive dendrites under clusters of short ribbons (Fig. 11a). Likewise, single confocal sections, showed PrPC intermingled among SCGN-positive cone bipolar cell dendrites (Fig. 11b). In infected mice at 104 and 118 dpi, staining with anti-PrP antibody D13 revealed an increase in PrPSc among SCGN-positive dendrites where they make synaptic connections with cone pedicles (Fig. 11a–f), and this deposition was coincident with the previous finding of PrPSc on the tips on *rod* bipolar cell dendrites beneath the horseshoe-shaped ribbon synapses present in rod photoreceptors (Fig. 9h–l). The timing and location of this increase in PrP staining suggested these deposits were likely to be the disease-associated form of PrP. Despite the destruction of most ribbon synapses in cones and the disappearance of cone bipolar cell dendrites, the number of SCGN-positive cone bipolar cells did not decrease during disease (Fig. 11g–k). Thus, both rods and cones were uniquely sensitive to prion-induced damage compared to other retinal neurons.

### Identification of PrPSc near horizontal cell dendrites and boutons

**Fig. 12:**
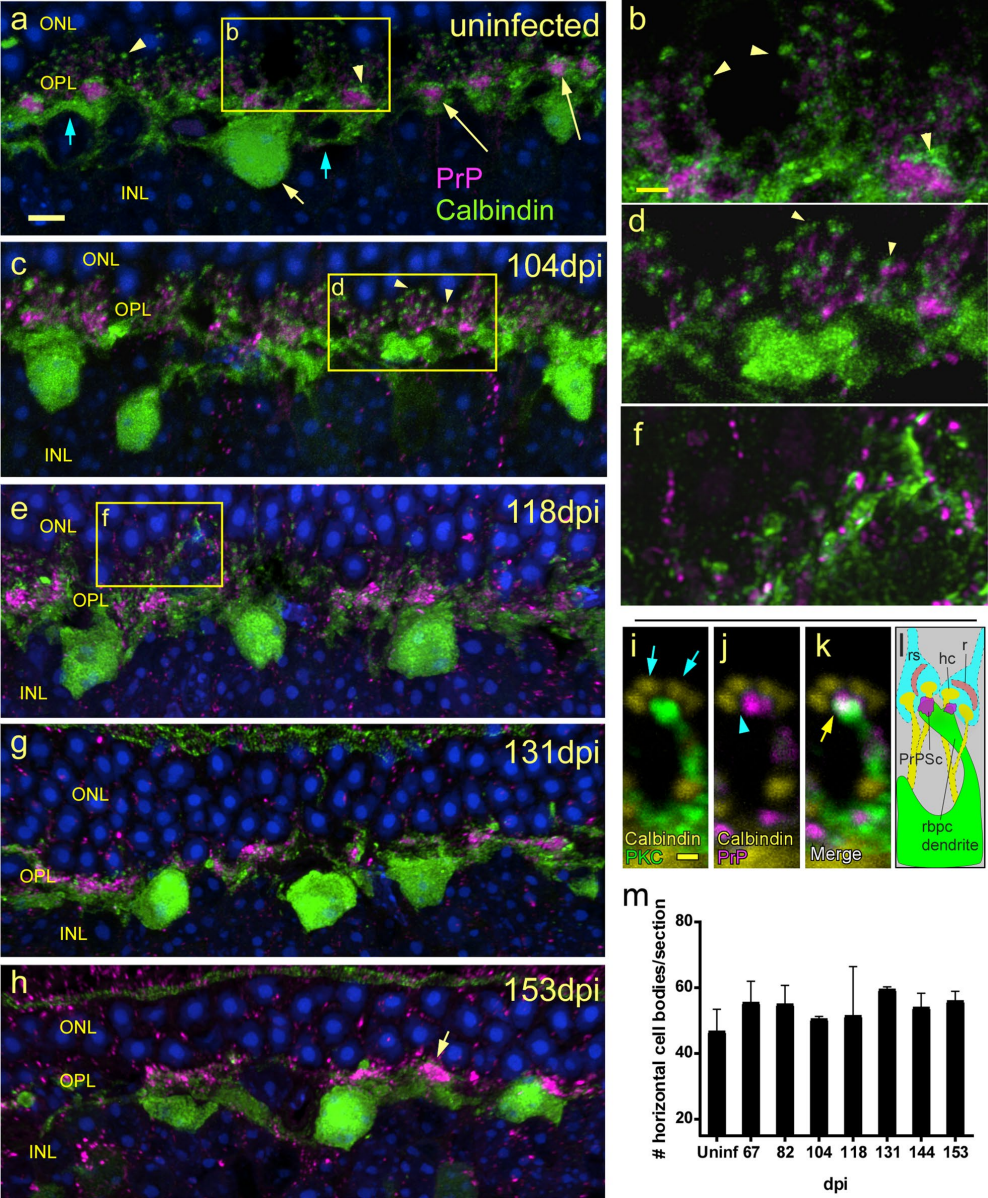
Timecourse and confocal analysis of PrP association with horizontal cells. **a,b** In uninfected mouse retina, anti-calbindin labels horizontal cell bodies (yellow arrow), dendrites (blue arrows) and boutons (arrowheads). PrPC magenta densities (long yellow arrow) are surrounded by horizontal cell boutons and dendrites. **c**, **d** At 104 dpi, calbindin-positive boutons (arrowheads) are near, but not touching, new PrPSc deposits (magenta). **e**, **f** At 118 dpi, PrPSc (magenta) is more widely distributed, and number of boutons is significantly reduced. PrPSc is near, but not in contact with horizontal cell components. **g**, **h** At later timepoints 131 and 153 dpi, ONL is thinned due to loss of photoreceptor nuclei. Few, if any, horizontal cell boutons are present. Large PrPSc deposits (arrow) are visible in the OPL, but not in horizontal cell bodies. **i** At very high magnification, a 118 dpi retina shows a PKCα-positive rod bipolar cell dendrite (green) near calbindin-positive (yellow) horizontal cell boutons (blue arrows). **j** A PrPSc deposit (magenta) is very close to, but not touching the horizontal cell boutons (blue arrowhead). **k** In a merge of three channels the white color indicates the association of a PrPSc deposit (magenta) with the dendritic tip of a rod bipolar cell (green). **l** Cartoon depicts preceding three panels, with addition of presumed location of rod spherules (rs) and ribbons (r). **m** Surprisingly, the number of horizontal cell bodies does not change significantly over time. Scale bars: **a**, **c**, **e**, **g**, **h** = 5 µm; **b**, **d**, **f** = 2 µm; **i–l = 0.5** µm

Along with dendritic processes from rod or cone bipolar cells, dendrites from two horizontal cell neurons (HC) make up the triad of processes that invaginate the photoreceptor at each ribbon synapse [36]. Therefore, HC were also studied during prion retinal infection using dual staining with anti-PrP and anti-calbindin, which is expressed on the cell body, dendrites and boutons of HC. In uninfected mice, HC bodies were vitread to the OPL, and processes and boutons were mostly in the OPL (Fig. 12a, b), and the clumped aggregates of PrP seen previously did not associate with either the HC or their processes. After scrapie infection at 104 dpi, HC bodies, processes, and boutons were unchanged (Fig. 12c, d). As expected, the PrP clumps seen previously in uninfected mice were missing, and PrP was now mostly disseminated in small punctate or linear deposits in the OPL which were near, but slightly separated from the HC boutons (Fig. 12c, d). This difference in PrP morphology suggested that this material was PrPSc. At 118 dpi, the HC boutons were less distinct and PrPSc was still similar to 104 dpi (Fig. 12e, f). There was still no association between HC bodies and PrP. This relationship was confirmed using confocal analysis of a triple stained section (Fig. 12i–l), where at high magnification, it was evident that calbindin-positive boutons did not contact PrPSc (Fig. 12j), whereas PrPSc was in contact with PKCα, the marker for rod bipolar cells (Fig. 12k). At 131 and 153 dpi the HC processes and the entire OPL region was atrophied, and damage appeared to be severe. However, PrPSc deposits were more consolidated and surprisingly, most HC bodies were intact (Fig. 12g, h, m).

In summary, at 118 dpi there was evidence of deposition of PrPSc in the OPL, at the ribbon synapses of rod photoreceptors. While PrPSc was not directly in contact with the presynaptic ribbons (CtBP2), it was associated with the postsynaptic elements of the synapses. PrPSc was found on the dendritic boutons of rod bipolar cells (PKCα), where they invaginated ribbons synapses and on cone bipolar cell dendrites (SCGN) adjacent to ribbon synapses. HC boutons (Calbindin) did not show accumulations of PrPSc. These data suggested that prion infection and PrPSc deposition on rod and cone bipolar cell processes might have a toxic effect on ribbon synapses leading to damage and death of both rods and cones.

### Electron microscopy of outer plexiform layer confirms loss of synapses

**Fig. 13:**
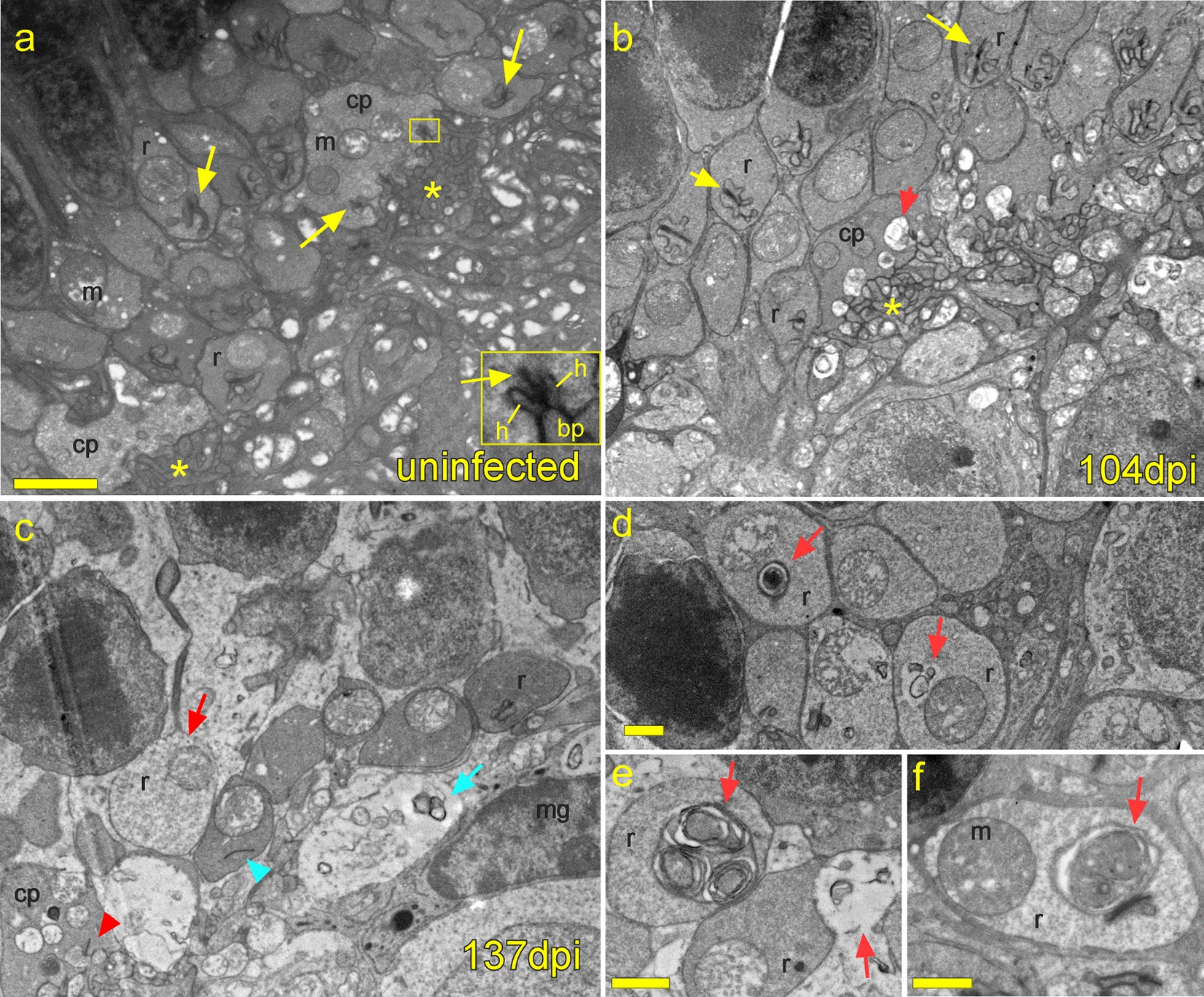
Transmission electron microscopy shows timeline of changes in outer plexiform layer. **a** Uninfected retina shows numerous rod spherules (r) each containing a single ribbon synapse (arrow) and mitochondrion (m). Cone pedicles (cp) contain multiple ribbon synapses (yellow arrows) and mitochondria (m). Inset shows magnified cone ribbon synapse with ribbon (arrow), and a typical triad consisting of two invaginating horizontal cell processes (h) and one bipolar cell process (bp). Many bipolar and horizontal cell processes are present vitread to cone pedicles (asterisks). **b** At 104 dpi, most rod spherules appear normal with ribbon synapses present. A cone pedicle (cp) at center, has swollen dystrophic dendritic processes at ribbon synapses (red arrow). Bipolar and horizontal cell processes are present beneath the pedicle (asterisk). **c** At 137 dpi the OPL appears disorganized with abnormal rod spherules (red arrow), a floating ribbon synapse (blue arrowhead) without invaginating dendritic processes, autophagic whorl (blue arrow) and a dystrophic dendrite (red arrowhead) at a ribbon synapse in a cone pedicle. Microglial cell (mg). **d**, **e**, **f** Examples of autophagic-like whorls (red arrows) in rod spherules at 137 dpi. Scale bars **a-c** = 2 µm; **d**, **e**, **f** = 1 µm

Analysis of the OPL by transmission electron microscopy confirmed many of the findings determined by confocal microscopy. TEM was advantageous in that it allowed a view of all structures simultaneously. Sections from uninfected mice showed many photoreceptor axon terminals (rod spherules and cone pedicles) containing ribbon synapses, most were connected to a triad of invaginating dendritic processes from horizontal and bipolar cells. Rod spherules contained a single large, round mitochondrion and a ribbon synapse, while cones contained multiple small mitochondria and 3–5 ribbon synapses (Fig. 13a). Early in disease (104 dpi), overall morphology was similar to uninfected mice (Fig. 13b), however in some cone pedicles, swollen, dystrophic dendritic processes were noted around ribbon synapses, suggesting that cone photoreceptors were beginning to degenerate (Fig. 13b). At later timepoints (Fig. 13c–f), typical triad ribbon synapses were rarely noted and both rod spherules and cone pedicles, if present, were often dystrophic. Frequent whorl structures, suggestive of autophagosomes, were noted adjacent to, or in place of ribbon synapses in both rods and cones. In short, these ultrastructural findings supported the conclusions from fluorescent microscopy that at late timepoints all elements of ribbon synapses had degenerated.

## Discussion

In the present study, we followed the timing and location of PrPSc deposition in retina following intracerebral prion injection in mice using immunofluorescence and electron microscopy. The earliest PrPSc deposits in retina were found at 67 dpi and were located in the IS region of cone photoreceptor cells. At 104 dpi PrPSc could be detected adjacent to the cilium, which connects the IS and OS portions of each cone and rod cell. These events were followed by cell swelling and alteration of organelles, such as rootlets, at 104 dpi, and progressive loss of cone and rod cell nuclei in the ONL starting at 118 dpi. Similar events with the exception of swelling were seen in rods on a time course about 14 days behind cones.

The association of PrPSc with cilia may be an important clue to the pathogenic process of prion infection in retina. This abnormal accumulation might interfere with or damage the ciliary protein transport system between the IS and OS regions of photoreceptors as has been proposed in retinitis pigmentosa and ciliopathies [34, 40]. In support of this mechanism, we found unusual distributions of opsin and cone arrestin (Figs. 4, 5, 6), suggesting interference with the normal trafficking of these proteins between the IS and OS.

Beginning at 104 dpi, abnormal PrPSc deposition was also detected in the OPL region near and within the cone pedicle and rod spherules. Although this process appeared to begin later than the process in the IS region, it still coincided with the detection of photoreceptor cell pathology at 104–118 dpi and thus might play a role in damage and death of rods and cones. In the OPL of uninfected mice, PrP was normally detected on or near the dendrites of cone and rod bipolar cells located vitread to rod spherules and cone pedicles, but after prion infection, PrP detection shifted to sites on the tips of bipolar cell dendritic boutons, where they invaginate rods to form ribbon synapses. Likewise, PrP deposits increased among the dendrites of cone bipolar cells where they synapse with cone pedicles. This different PrP distribution suggested that this material was disease-associated PrPSc. During this same time, starting at 104–118 dpi and continuing up through 153 dpi, there was progressive loss of ribbons in rods and cones associated with loss of over 90% of photoreceptor cone and rod nuclei in the ONL. The mechanism of damage induced by the presence of PrPSc on the tips of bipolar cell dendritic boutons is not clear. However, the accumulation of protein aggregates at sites of synaptic transmission might confound the synaptic transmission process or alter neuritic connectivity [53, 61]. Similar speculations have been suggested for hippocampal synapses in prion-infected brain [12, 27, 48]. However, this has not previously been suggested for retinal photoreceptor ribbon synapses, where the molecular components can be visualized in better detail.

Interestingly other nearby retinal neurons were damaged less by prion infection. For example, cell bodies of cone bipolar cells remained constant throughout disease, rod bipolar cell bodies decreased by 50%, and horizontal cell bodies decreased less than 10%. However, dendrites in the outer plexiform layer of all these cells showed significant damage. Ganglion cells and amacrine cells also did not appear to be damaged. These latter two cell types were not specifically stained and counted, but cellularity in the regions where they typically reside appeared to be normal. One explanation for the extreme sensitivity of cone and rod cells to prion infection might be the PrPSc association and blockage of cilia which are not found in the other above-mentioned retinal neurons. Alternatively, the level of PrPC expression in various cell types might also play a role in these differences. In contrast, photoreceptor degeneration was not observed in our studies of prion-infected transgenic mice expressing GPI-anchorless PrP, suggesting expression of anchored PrPC on the cell membrane may be critical to PrPSc-induced damage to certain cell types [30].

Another question raised by these results is why the cones preceded rods in the PrPSc deposition and damage process. In some rare retinal degenerations, known as Cone and Cone-rod dystrophies, cones also precede rods in damage and death, however it is more common for rods to begin the degenerative process, as in retinitis pigmentosa [39, 56]. In other neurodegenerative diseases affecting retina, such as AD and PD, photoreceptors are not typically affected first and specificity for cones or rods is not clear, though a recent study found accumulations of phosphorylated tau specifically in ageing primate cones [1, 35, 44]. Interestingly, cones make up only 3% of photoreceptors in mouse retina, so the cone cell specificity in our study was surprising. This could be due to local differences in PrP expression which might influence the timing of PrP conversion to PrPSc. We have not been able to measure differences in normal PrP expression in the IS region of uninfected rods and cones. However, in the OPL region, uninfected mice have high PrPC expression in the dendritic processes of bipolar cells located directly beneath the cone pedicles. These are a mixture of rod and cone bipolar cells which all appear to deposit PrPSc on their dendrites after prion infection, but while there are hundreds of synaptic connections to each cone pedicle, each rod spherule has no more than seven [38]. This difference might increase the efficiency of prion travel to the IS regions of cones vs rods. In addition, prion travel from ganglion cells to cone cells can be direct, whereas intermediate cells, such as amacrine cells, are often involved in connecting ganglion cells to rod bipolar cells [14]. The more direct route to cones might increase the tempo of prion infection, favoring cones over rods.

The damage mechanism(s) seen in prion-infected retina and brain are not well understood [25]. Our earlier studies indicated the presence of apoptotic cells in the ONL after prion infection [29, 54]. Since these observations were made later in the disease course, these cells were likely to be mostly rods which are 32 × more abundant than cones in mouse retina. However, in our studies using cone opsin to detect cones, damaged PrPSc-positive cones in the IS region at 104 and 118 dpi were swollen suggesting a necrotic rather than apoptotic cell death process (Figs. 4, 5, 13). Interestingly, this same pathology preceding cone necrosis is similar to previous results of Murikami et al. [37] studying the human retinitis pigmentosa rd10 mutation in a mouse model and in human retinitis pigmentosa patients.

## Conclusions

The present experiments report two new areas of deposition of abnormal disease-associated PrPSc in prion-infected retina. These regions both involve photoreceptor cone and rod cells which then go on to die as a part of the disease process. The location of the PrPSc deposition suggests that the damage mechanisms may involve interruption of the ciliary transport pathways of molecules between the inner and outer segments of PR cells as well as damage to ribbon synapses found in the synaptic end-feet of rods and cones near the OPL. Possible synergy and specificity between these two mechanisms remains to be worked out. These mechanisms might be active in other human retinal degenerative diseases where protein misfolding occurs, such as retinitis pigmentosa and prion-like diseases, such as AD and PD.

## Supplementary information

**Additional file 1: Fig. 1** Cone pedicles and rod spherules disappear as PrPSc accumulates in the OPL. a In an uninfected mouse, concentrations of PrPC (magenta, yellow arrows) lie vitread to cone pedicles (dark space, yellow arrowhead) outlined by glucose transporter 1 (GLUT1) a membrane-associated protein. Rod spherules (red arrowhead) often have tiny faint patches of PrPC (red arrow) adjacent to their membranes. b At 118 dpi, cone pedicles are less frequent and the distribution of PrP is more punctate and widespread, suggesting PrPSc deposits. c at 153 dpi OPL has thinned significantly, most rod spherules have disappeared, and PrPSc is now in dense punctate patches. ONL has thinned to 1-2 nuclei. Scale bar = 5 µm. a, b, c are images of optical slices with total z-depth =1 µm

## Data Availability

The data supporting the conclusions of this article are included within the article. Original slides, tissues and photographs are retained. All reagents and animals used in this study are available from scientific supply companies, except the anti-PrP antibody D13, which depending on supply, may be available upon request.
