# Supplementary figures and images for: Prion-induced photoreceptor degeneration begins with misfolded prion protein accumulation in cones at two distinct sites: cilia and ribbon synapses
Source: Acta Neuropathol Commun. 2021 Jan 29;9:17. doi: 10.1186/s40478-021-01120-x (PMC7845122; doi:10.1186/s40478-021-01120-x)

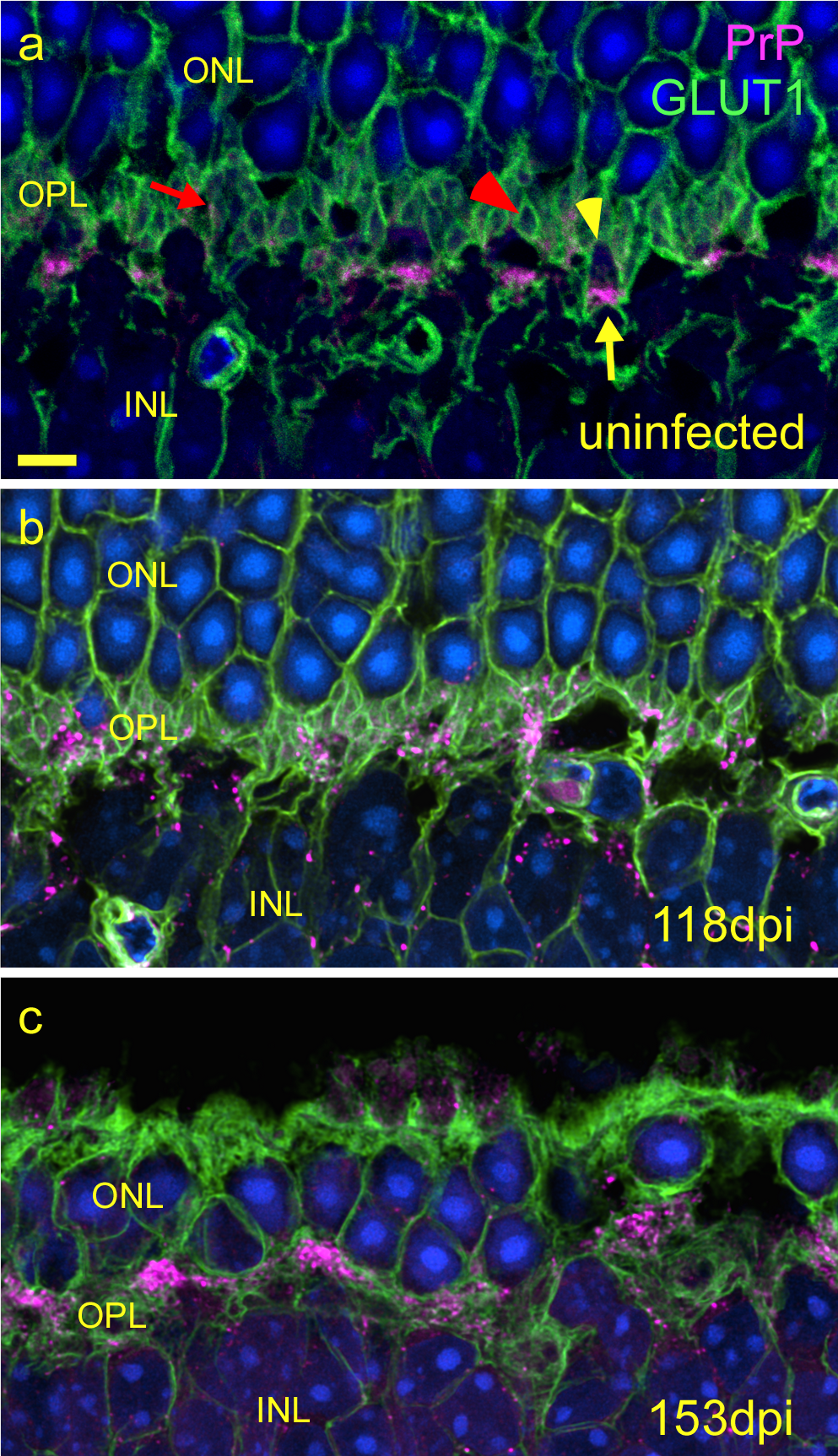

Supplement: Supplementary file 1 — Additional file 1: Fig. 1 Cone pedicles and rod spherules disappear as PrPSc accumulates in the OPL. a In an uninfected mouse, concentrations of PrPC (magenta, yellow arrows) lie vitread to cone pedicles (dark space, yellow arrowhead) outlined by glucose transporter 1 (GLUT1) a membrane-associated protein. Rod spherules (red arrowhead) often have tiny faint patches of PrPC (red arrow) adjacent to their membranes. b At 118 dpi, cone pedicles are less frequent and the distribution of PrP is more punctate and widespread, suggesting PrPSc deposits. c at 153 dpi OPL has thinned significantly, most rod spherules have disappeared, and PrPSc is now in dense punctate patches. ONL has thinned to 1-2 nuclei. Scale bar = 5 µm. a, b, c are images of optical slices with total z-depth =1 µm [file 40478_2021_1120_MOESM1_ESM.tiff]
